# Rock Surface Fungi in Deep Continental Biosphere—Exploration of Microbial Community Formation with Subsurface In Situ Biofilm Trap

**DOI:** 10.3390/microorganisms9010064

**Published:** 2020-12-29

**Authors:** Maija Nuppunen-Puputti, Riikka Kietäväinen, Lotta Purkamo, Pauliina Rajala, Merja Itävaara, Ilmo Kukkonen, Malin Bomberg

**Affiliations:** 1 VTT Technical Research Centre of Finland Ltd., 02044 Espoo, Finland; pauliina.rajala@vtt.fi (P.R.); merja.itavaara@gmail.com (M.I.); malin.bomberg@vtt.fi (M.B.); 2 Geological Survey of Finland, 02151 Espoo, Finland; riikka.kietavainen@gtk.fi (R.K.); lotta.purkamo@gtk.fi (L.P.); 3 Physics Department, University of Helsinki, 00014 Helsinki, Finland; ilmo.kukkonen@helsinki.fi

**Keywords:** crystalline bedrock, in situ sampling, saline groundwater, terrestrial deep subsurface, deep subsurface fungi, ICDP

## Abstract

Fungi have an important role in nutrient cycling in most ecosystems on Earth, yet their ecology and functionality in deep continental subsurface remain unknown. Here, we report the first observations of active fungal colonization of mica schist in the deep continental biosphere and the ability of deep subsurface fungi to attach to rock surfaces under in situ conditions in groundwater at 500 and 967 m depth in Precambrian bedrock. We present an in situ subsurface biofilm trap, designed to reveal sessile microbial communities on rock surface in deep continental groundwater, using Outokumpu Deep Drill Hole, in eastern Finland, as a test site. The observed fungal phyla in Outokumpu subsurface were Basidiomycota, Ascomycota, and Mortierellomycota. In addition, significant proportion of the community represented unclassified Fungi. Sessile fungal communities on mica schist surfaces differed from the planktic fungal communities. The main bacterial phyla were Firmicutes, Proteobacteria, and Actinobacteriota. Biofilm formation on rock surfaces is a slow process and our results indicate that fungal and bacterial communities dominate the early surface attachment process, when pristine mineral surfaces are exposed to deep subsurface ecosystems. Various fungi showed statistically significant cross-kingdom correlation with both thiosulfate and sulfate reducing bacteria, e.g., SRB2 with fungi *Debaryomyces hansenii.*

## 1. Introduction

The deep continental subsurface supports diverse microbial life, comprising up to 20% of Earth’s biomass [1,2]. Fungi are adapted inhabitants of rock surfaces, and both endolithic and epilithic fungi are found from rocks even from the most extreme living conditions [3,4,5,6]. The anoxic deep continental bedrock environment is a hostile and challenging, oligotrophic habitat for microorganisms. Deep biosphere microbial communities reside either as planktic communities in deep groundwater or sessile communities on surfaces [7,8,9]. The formation of biofilms and microcolonies on fracture zone rock surfaces has been demonstrated in various deep subsurface settings, e.g., gold mines and deep subseafloor crust [10,11,12,13,14,15,16]. Drake et al. (2017, 2018) recently revealed fossil fungal cryptoendolithic communities with hyphae-like structures from deep drill core rock surfaces, thus linking fungi to deep subsurface biofilms [17,18]. Small fossilized yeast-like structures have also been visualized in association with bacterial biofilms in Triberg granite, Germany [19].

Microbial attachment on rock surfaces enables both biochemical and biomechanical microbe–mineral interaction potentially leading to dissolution of minerals [20,21]. Further formation of attached community could also advance syntrophic/mutualistic interaction between species. Rock surface communities potentially represent taxa capable of exploiting mineral material as their energy and/or carbon and nutrient source [22,23]. First, a thin conditioning film containing various organic compounds is formed on surfaces exposed to deep groundwater, which then enables microbial cell attachment [24,25]. Genes linked to biofilm formation, such as those for extracellular polymeric substance (EPS) formation and secretion, have been detected from deep subsurface biofilm metagenomes [26]. Furthermore, visualization of extracellular polysaccharides, proteins, and lipids in multi-species deep subsurface biofilms further support the claim that active microbial communities form biofilms on rock surfaces despite its energetic costs [27].

In situ flow-through reactors are commonly used in deep subsurface biofilm research [23,28,29]. For example, Casar et al. (2020) showed that host minerals have potential to increase the abundance of rock-surface attaching cells, modify community structure, and advance especially extracellular electron transfer utilizing microbial communities in flow-through reactors [23]. Another option for in situ sampling is acquisition of deep drill cores with fresh biofilms [12,30,31]. However, microbiological sampling of deep subsurface drill cores is possible only during initial drilling operation as targeted drilling at different depths cannot be accomplished similarly to those of deep subsurface facilities, such as the Äspö Hard Rock Laboratory (HRL) in Sweden or the Sanford Underground Research Facility (SURF) in the USA [12,30]. In comparison to flow cells, Jägevall et al. (2011) showed that microbial community composition of the natural biofilms attached to deep crystalline bedrock aquifer surfaces differed from the biofilm that developed on glass and rock surfaces in laminar flow-cells [12]. Observed differences between naturally formed and flow-cell harvested microbial biofilm communities demonstrate the need to further develop in situ applications. Most flow-through reactors are not applicable in deep drill hole environments. Instead, biofilm formation in deep drill hole should be studied e.g., in packer-isolated borehole segments, as demonstrated by Amano et al. (2017) [32]. Their 10-year in situ incubation study showed that the biofilms developed on teflon tubings hosted very low microbial community diversity which also differed from the deep planktic groundwater communities [32]. As there are differences in the microbiology of biofilms and related groundwater, and fracture surfaces cannot be sampled in situ, biofilm traps can be applied as a simulation of the fracture surface attached microbial life forms.

Besides more comprehensively studied Bacteria and Archaea, also less abundant Eukaryotes have been shown to be part of the deep biosphere [33,34,35,36,37,38,39,40,41,42,43,44]. Deep subsurface fungi have smaller average cell size compared to known yeasts [33]. Sohlberg et al. (2015) were the first to describe highly diverse and active communities of deep groundwater fungi in Olkiluoto, Finland [35,42]. A large part of the deep biosphere fungal communities affiliate with unclassified, uncultured taxa, thus their functionality remains understudied. Despite the knowledge on community structure of deep groundwater fungi, there is very limited information on the rock surface fungal communities and their role in biofilms. In soil, fungi are the key organisms linked to the leaching of nutrients from soil minerals to their symbiotic or mutualistic partner organisms, such as trees, in exchange for carbon compounds [45]. Whether they hold a similar symbiotic role in the deep biosphere and leach nutrients from the surrounding rock for other microbial groups is still poorly understood. As heterotrophs, fungi depend on the other microbial groups for their carbon in deep bedrock. Indeed, Drake et al. (2017) described a conceptual model for symbiotic processes between deep subsurface fungi, sulfate reducing bacteria (SRB), and anaerobic methane oxidizing communities (AOM), in which the fungi would receive organic carbon from the biofilm and in turn provide SRB with H_2_ [17]. Thus, fungi could have an important role in nutrient cycling in the deep continental subsurface.

In order to describe the microbial community structure of the attached microbial population, incubations carried out in situ in natural environments have an advantage. This way, the indigenous microbial communities at the targeted study depth are not affected by pressure drops, changes in concentrations of soluble gases, or nutrient levels over the incubation period. Our specific aims of this project were to study the rock-attached microbiomes with special focus on fungal communities, and to show biofilm formation at in situ conditions in deep bedrock. We developed a biofilm incubation chamber (trap) that enables both sampling and in situ incubation in deep bedrock groundwater. Our study site in Finland, the Outokumpu Deep Drill Hole, spans several fluid-filled fracture zones and reaches the final depth of 2.5 km [46,47]. The fluids are anoxic and saline, and the salinity increases with depth. Previous studies showed diverse microbial communities in groundwater with especially rich and active communities of SRB at the depths of 500 and 967 m [48,49,50,51]. These depths contain major fractures with fluid flow into the drill hole, making them ideal for our in situ incubation study [50]. The trap model was designed to elucidate microbial communities attached to different surfaces (mica schist and glass) and allow comparison to planktic microbial communities detected in the surrounding fluid. Mica schist is found throughout the lithological profile of the Outokumpu Deep Drill Hole, and it was chosen for this study since it is the main rock type at the incubation depths and the indigenous microbes were adapted to this rock type. The Outokumpu mica schist is a metamorphic rock type derived from sediments deposited on the continental shelf and deep sea and metamorphosed during the Precambrian in the Svecokarelian orogeny. Metamorphic peak conditions at about 1.9 Ga ago were 8 kbar and 670 °C [52]. Outokumpu mica schist’s main minerals are quartz, plagioclase, and biotite, and it contains small amounts of carbon, sulfur, phosphorus, and iron [53]. Therefore, it also represents a potential substrate, carbon and nutrient source for the microorganisms.

## 2. Materials and Methods

### 2.1. Site Description

Outokumpu scientific drill hole is located in eastern Finland (62.72° N, 29.07° E), and it pierces through Precambrian bedrock mainly consisting of metamorphosed sediments and granitic rocks [53]. The Outokumpu Deep Drill Hole is part of the International Continental Scientific Drilling Program (ICDP) infrastructure, https://www.icdp-online.org/projects/world/europe/outokumpu-finland/. Previous studies have described the site and its hydrogeochemical characteristics [46,47,54,55,56,57], and microbial community structure and functionality in the fracture fluids [35,48,49,51,58,59]. The lithological profile of the Outokumpu Deep Drill Hole has been previously described and mica schist is the prevalent rock type at our incubation depths [53,60]. However, immediately above the 500 m sampling depth, the main rock type changes to chlorite-sericite schist, a hydrothermally altered variant of the common biotite dominated mica schist. The groundwater table is typically approximately at 10 m below the ground surface. Water type at both sampling depths is Na-Ca-Cl, with dominating Cl^−^ concentration above 7000 mg L^−1^ [55,56]. The major gas component of the fluid is CH_4_, with 22 and 32 mmol L^−1^ at 500 and 967 m depth, respectively [56].

### 2.2. Description of the In Situ Biofilm Trap

**Figure 1:**
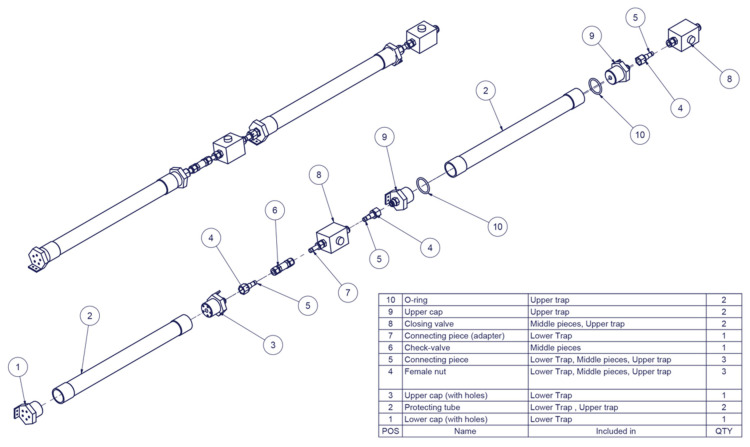
Schematic diagram of the in situ biofilm trap. More details are shown in Figures S1 and S2.

The in situ biofilm trap was designed to enable observation of biofilm formation and characterization of attached and planktic groundwater microbial communities. The biofilm trap was manufactured from robust materials, mainly stainless steel, which enabled proper sterilization prior to application, resistance to high hydrostatic pressure up to 100 bar, and corrosion resistance. The schematic diagram of the biofilm trap shows the two-part structure (Figure 1, Supplementary Materials Figures S1 and S2). Each trap unit contains both an upper, closed trap for in situ incubation, and a lower, open trap enabling exchange of fluid during the incubation period. The in situ biofilm trap was designed to enable pumping of the fracture fluids through the trap prior to the incubation period. In addition, a backpressure valve at the lower end of the closed trap enables fluid sampling at the end of the incubation. The biofilm traps were connected with polyamide (PA) tubings (10/12 mm diam.), allowing pumping of water through the traps and to the surface. A submersible membrane pump at the depth of 35 m was applied. The traps and the tubings were supported in the well with a 5 mm diameter stainless steel (AISI316) cable operated with a drill hole winch.

In the experimental setup, we used two separate biofilm traps with separate PA-tubings (10/12 mm in diameter), one set for each incubation depth, 500 and 967 m. Three separate sample cages were inserted into each in situ biofilm trap cabinet, one for glass microscopy slides (25 × 75 × 1 mm) (as control surface material), one for mica schist slides, and one for crushed mica schist (Figure S2). Mica schist slides and crushed rock were prepared at the Geological Survey of Finland in Espoo from the Outokumpu Deep Drill Core resembling the corresponding depth. The 38 × 16 × 4 (mm) sized slides were first cut with a rock saw, then polished and washed in an ultrasonic bath. Rock crush was prepared in a ball mill and the small size fraction (<5 mm) was separated by sieving and used in the experiment. Once filled, the sample cages were subsequently heat sterilized over-night at 160 °C. The trap cabinets were then prepared by inserting the separate cages into the trap, cages containing rocks in the bottom, followed by autoclaving (dry program, 121 °C, 15 min). The small parts of the trap were packed into aluminum foil and autoclaved as the trap cabinets. The separately autoclaved parts and two cabinets were assembled in the field laboratory. During assembly, the trap was handled with latex gloves and exposed surfaces were disinfected with 70% ethanol. The traps were attached to the metal cable and PA-tubings at the drill hole, only right before lowering, and further secured around the trap cabins with duct tape to ensure the attachment to the cable.

### 2.3. Application of the In Situ Biofilm Trap and Sampling

The six-month in situ incubation was carried out between November 2010 and May 2011. Two sets of traps and PA-tubings were lowered to the incubation depths of 500 and 967 m located at known fracture zones with fluid in-flow to the drill hole. When the biofilm traps reached the desired depths, 135 and 95 L of water was first pumped through the 967 and 500 m trap, respectively, using a membrane pump, in order to purge the fracture zone areas of mixed fluids from the descent of the traps. EC, pH, dissolved O_2_, and redox-potential were monitored during the pumping in a flow-through cell equipped with suitable sensors (WTW and Hamilton). A platinum (Pt) electrode was used for the measurement of the redox-potential, and the measured values corrected for the reference electrode’s voltage (+207 mV) relative to the standard hydrogen electrode (SHE). (Figure S3). In addition, hydrogeochemical samples were taken from both depths, after the monitored parameters had stabilized sufficiently.

The biofilm traps were removed from the incubation depths in May 2011 after 6 months of deployment. When lifted from the drill hole, the traps were instantly protected with N_2_ gas flow, which was led to the part of the capped drill hole above the fluid level. After removal from the drill hole, the trap was immediately placed into an anaerobic glove bag (AtmosBag^®^, Sigma-Aldrich, St. Louis, MO, USA). N_2_ atmosphere was created in the glove bag by purging the glove bag using a vacuum pump and replacing gas with N_2_, the purging cycle was repeated three times. In addition, residual oxygen was removed chemically using anaerobic generators (Anaerocult A, Merck, Darmstadt, Germany) and the glove bag was kept under constant low N_2_ flow to protect the open part of the trap. The trap enclosed in the anaerobic glove bag was then moved into an anaerobic glove box (MBRAUN, Garching, Germany) for further processing. Because of the size of the trap, it could not be inserted into the glove box while maintaining an anaerobic atmosphere in the box. Thus, the box interior was rendered anaerobic by constant N_2_ gas flow through the box for three hours in addition to the use of anaerobic generators inside the glove box. When the oxygen was purged from the glove box, the trap was removed from the protective anaerobic glove bag.

The biofilm trap was opened, and water and biofilm samples were collected for further analysis (Table 1). First, fluid samples of 650 and 700 mL were decanted from the closed traps into acid-washed sterile Schott-bottles (Duran Group, Wertheim/Main, Germany). For nucleic acid extraction, biomass of duplicate 100 mL (500 m) or 50 mL (967 m) subsamples were collected on 0.22 µm pore-sized Sterivex filters (Millipore, Billerica, MA, USA) (Table 1). Similarly, crushed mica schist samples were collected from the sample cages in sterile Corning centrifuge tubes (Corning Inc., New York, NY, USA), and transported on dry ice to the laboratory and frozen at −80 °C prior to DNA extraction. Water samples for geochemical analysis were taken from the closed traps. For cation analyses, 100 mL water samples were filtrated through 0.22 µm filter (Whatman, GE Healthcare UK Ltd., Buckinghamshire, UK) into acid washed plastic bottles amended with 0.5 mL of HNO_3_ (65%) and kept at +4 °C until analysis. In addition, 60 mL filtrated fluid sample (0.22 µm filter, Whatman, GE Healthcare UK Ltd., Buckinghamshire, UK) was collected for water stable isotope analysis and 150 mL unfiltrated sample for anion analysis from each depth. Samples for scanning electron microscopy (SEM) (mica schist slides and glass) were fixed immediately after sampling at the field laboratory with glutaraldehyde (Table 1). In detail, 5 mL of glutaraldehyde was mixed with 45 mL of fracture fluid to achieve final 2.5% glutaraldehyde concentration, further added to mica schist slide samples and transferred to 50 mL centrifuge tubes. Samples were stored at +4 °C until SEM-analysis. Duplicate 60 mL fluid samples were collected for cell counting from both closed traps to headspace bottles and stored at +4 °C until analysis.

### 2.4. Cell Count

Enumeration of the total cell counts in fluid samples was performed as previously described [38,50]. Duplicate fluid samples (5 mL) from closed traps for both sampling depths were dyed with 50 µL of 4′-6-diamidino-2-phenylindole (DAPI) stain (2.5 mg mL^−1^ in 2.5% glutaraldehyde) (Sigma-Aldrich, St. Louis, MO, USA) for 20 min in darkness. Then, DAPI-stained samples were filtrated onto black 0.2 µm GTBP- filters (Millipore, USA) with a low-vacuum filtration unit (Millipore) and placed onto microscopy slides prior to epifluorescence microscopy analysis. Samples were analyzed with an Olympus B×60 microscope (Olympus Optical Ltd., Tokyo, Japan) and thirty random fields were viewed and counted in order to determine the total cell count.

### 2.5. Geochemical Analysis of Water Samples

In addition to the measurements of geochemical parameters on-line during flushing of the traps in November 2010, geochemical parameters were determined in the laboratory for samples collected both before and after the incubation experiment. Most analyses were done by Labtium Oy (Espoo, Finland). Cations were determined with ICP-MS and ICP-OES techniques and anions with ion chromatography according the SFS-EN-ISO 10304-1 standard. Alkalinity was determined by end point titration to pH 4.5, and EC determined with a potentiometric method. Water stable isotope analysis was conducted at the Geological Survey of Finland in Espoo using a cavity ring-down spectroscopy (CRDS)-based water analyzer (Picarro, Santa Clara, CA, USA) (Table S1).

### 2.6. SEM Measurements for Mineralogy

Mica schist slides, placed in the 500 m trap, were imaged with scanning electron microscope (SEM, JEOL JSM-5900) at the Geological Survey of Finland in Espoo, prior to and after the experiment. Low vacuum, energy dispersive X-ray spectroscopy (EDS) mode with acceleration voltage of 20 kV and spot size of 50 µm was used for qualitative analysis of chemical composition of the uncoated rock samples. No slides placed in the 967 m trap, or crushed rock, were analyzed, but similar mica schist was used in all traps.

### 2.7. SEM Measurements for Microbiology

Mica schist slides that were incubated in situ for 6 months at 500 and 967 m depths, were fixed with glutaraldehyde (2.5% final concentration) in the field. The fixed samples were dehydrated with an ethanol series (30%, 50%, 70%, 80%, 96%, and absolute EtOH), followed by a final desiccation step with hexamethyldisilazane (HMDS) prior to SEM-analysis in the laboratory. The mica schist slides were coated with Au/Pd. FE-SEM examination was performed with Hitachi S-4800 FE-SEM at the University of Helsinki.

### 2.8. DNA Extraction

Triplicate mica schist crush samples and single glass samples were used for DNA extraction to characterize the microbial community forming biofilm on the surfaces (Table 1). First, 4 g of crushed rock/half of a glass slide and 10 mL of 1× PBS containing 1 µL mL^−1^ Tween 20 (Bio-Rad, Hercules, CA, USA) was added into 50 mL centrifuge tube (Corning Inc., NY, USA), followed by shaking (10 min at 150 rpm), sonication (3 min) and finally detached biomass was collected on 0.22 µm Sterivex filter units (Millipore, Burlington, MA, USA). Duplicate water samples of the closed trap fluid were filtrated on Sterivex filters already in the field glove box in order to collect the planktic biomass. Filters were cut in half with a sterile scalpel. DNA was extracted from the filter halves with the PowerWater DNA isolation kit (MoBio Laboratories, Inc., Carlsbad, CA, USA). Both the filter and the processed crushed mica schist of each sample was added to the corresponding extraction tube in order to lyse also the cells possibly remaining on the rock surface after the detachment process. DNA extraction was performed according to the manufacturers protocol and DNA was eluted in 50 µL of molecular grade water. Negative reagent controls for DNA extraction were handled similarly to sample extractions. DNA concentrations were measured with the Nanodrop-1000 spectrophotometer (NanoDrop Technologies Inc., Wilmington, DE, USA).

### 2.9. Amplicon Sequencing

Samples were prepared for IonTorrent amplicon sequencing (fungi, bacteria, archaea) as described in Bomberg et al. (2017) and Purkamo et al. (2017) [35,61]. The fungal primers targeted the ITS1 region and the 16S rRNA gene primers targeted V3-4 region and V4 region for bacteria and archaea, respectively [62,63,64]. The amplifications were done in two separate 25 µL PCR reactions per sample using the MyTaq RED Mix (Bioline, UK). The template volume was 4 µL and 20 pmol of forward and reverse primers were used in each reaction. All amplicon barcoding PCRs were performed in 96-well PCR plates (4titude Ltd., Surrey, UK) with an Eppendorf Mastercycler Nexus (Eppendorf, Hamburg, Germany). The amplification was performed with initial denaturation at 95 °C for 3 min, followed by 40 amplification cycles of 15 s at 95 °C, 15 s at 50 °C, and 15 s at 72 °C, and a final elongation at 72 °C for 30 s. Amplicon products were checked with agarose electrophoresis. IonTorrent PGM platform amplicon sequencing was performed at Bioser (Oulu, Finland) with the 316 Chip Kit v2 and kits for Ion PGM Template IA 500 and Ion PGM Hi-Q Sequencing (Thermo Fisher Scientific, Waltham, MA, USA). Negative control samples for the DNA extraction control and amplicon barcoding PCR were included in the sequencing set.

### 2.10. Sequence Data Handling and Statistical Analysis

Amplicon sequencing data were analyzed with the mothur software version 1.43.0 [65]. First, the sequence data was demultiplexed and quality checked with parameters of pdiffs = 2 for bacteria and fungi, pdiffs = 3 for archaea, bdiffs = 0, qwindowaverage = 20, qwindowsize = 50, and maximum amount of homopolymers was 8. The minimum sequence length was 180 bp for all amplicon types. Un-aligned fungal ITS1 sequences were clustered with the acg algorithm, whereas bacterial and archaeal sequence reads were aligned against the Silva.nr.v138 with targeted gene area alignment as pcr.seqs optimization [66,67]. Taxonomy for clustered sequences was assigned using the UNITEv8 reference database for fungi and the Silva.nr.v138 reference database for bacteria and archaea using the wang algorithm with 80% cut-off [68,69]. Sequence reads obtaining unspecific taxonomical assignments, e.g., non-bacterial sequences in the bacterial dataset, were removed. Clustered sequences were checked for chimeras with the chimera.vsearch, thereafter chimeric sequences were removed, and the remaining clustered sequences were assigned to OTUs (Operational Taxonomic Units) based on 97% within OTU sequence similarity. A .biom file was constructed with mothur and was transferred to the Microbiome Helper, where the data were further processed with Qiime [70,71]. OTUs detected in the negative control samples were carefully evaluated and filtered out of the sample data, unless they were present in higher abundance in the actual samples compared to controls and were regarded as potential authentic subsurface taxa. For the fungal dataset, OTUs with max 4 sequences in the negative controls combined with over 100 sequences in the samples were kept, and similarly for bacteria, OTUs with max 8 sequences in negative controls combined with over 100 sequences in the samples were kept. Cleaned data were re-introduced to Qiime and new .biom file was constructed. Archaea were not detected in all sample types, observed number of sequences was low, and thus archaeal data were not analyzed further.

Statistical data analysis was performed with R v. 3.6.2 in RStudio [72]. The microbial community alpha and beta diversity was analyzed with packages vegan and phyloseq [73,74]. Data visualizations were conducted with package ggplot2 [75]. Similarities/dissimilarities of microbial community composition of trap fluid, mica schist rock and glass surface samples were compared with principal coordinate analysis (PCoA) with relative abundance data and Bray–Curtis dissimilarity model in phyloseq. Alpha-diversity measures were calculated from raw read counts with the estimate_richness function in phyloseq. Cross-kingdom OTU correlations were done with data filtered to contain only OTUs that were present in at least two rock surface samples and contained a raw read minimum of 50 for fungi and 20 for bacteria. Data were manually reformed into a mothur .shared file by adding columns for label (0.03) and number of OTUs (numOtus), and further analyzed with the sparcc command in mothur. The correlation and *p*-value matrix were imported to R and visualized with package ggcorrplot.

### 2.11. Accession Numbers and Data Availability

The IonTorrent sequences in this study were deposited in European Nucleotide Archive (ENA) under project PRJEB40820 and accession numbers ERS5208985-ERS5209024.

## 3. Results

### 3.1. Cell Count and DNA Concentration

We retrieved samples from two different trap models from 500 and 967 m depths for molecular biology, SEM, and cell counting purposes (see Table 1 for detailed scheme for samples). These samples had been incubated for 6 months under in situ conditions. Cell counts in the fluid samples were 3.8 × 10^5^ cells mL^−1^ and 2.5 × 10^5^ cells mL^−1^, in the 500 and 967 m closed traps, respectively. Measured DNA-concentrations in all samples varied between 0.9 to 27 ng µL^−1^.

### 3.2. Biogeochemistry of the Trap Fluids

The geochemical data shows that an isolated microcosm environment had formed within the biofilm traps (Table 2). The pH had dropped from 9.6 to 6.4 and from 9.9 to 6.7 in the closed biofilm traps at 500 and 967 m, respectively. The electrical conductivity (EC) had dropped from 1940 to 1800 mS m^−1^ (at 25 °C) at 500 m depth and from 2030 to 1840 mS m^−1^ (at 25 °C) at 967 m depth. In addition, fluid alkalinity had increased from approximately 0.2 to over 0.63 mmol L^−1^. Interestingly, increase of elemental Fe, P, and S concentrations were detected in the trap fluids compared to the original fracture fluids. Similarly, increased levels of various trace elements were observed in the trap fluid samples (Table S1).

### 3.3. Imaging Rock Surface Attached Microbial Cells by SEM

**Figure 2:**
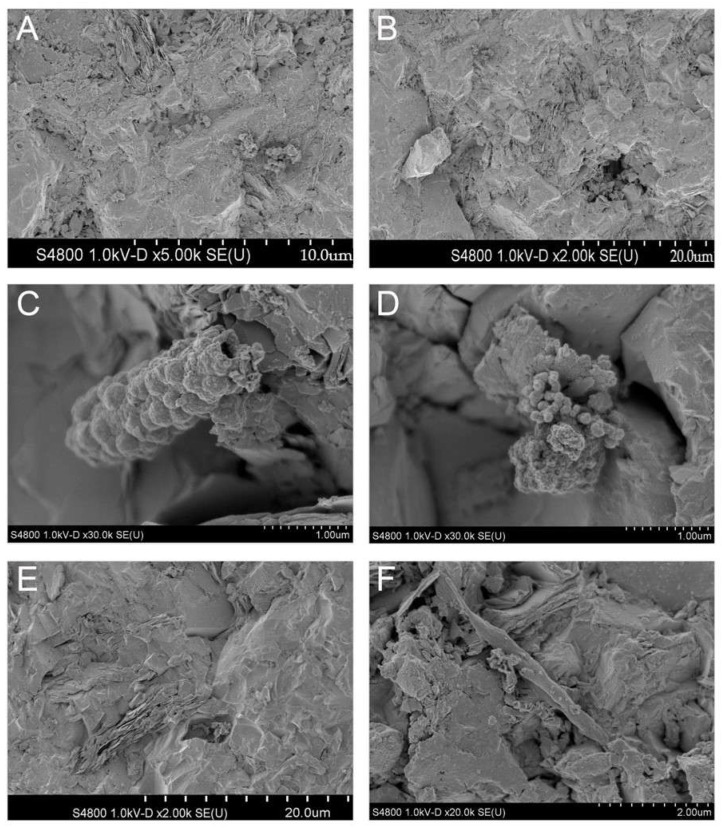
SEM analysis of the in situ incubated mica schist surfaces from the closed trap model (**A**,**B**) and open trap model (**C**–**F**) from the depth of 500 m. Each figure has a scale at the bottom for size reference (um = μm). (**A**) Various potential partly dehydrated microbial cell structures, on the left potential coccoid cells, and on the right cell-like clusters. (**B**,**E**) Microbial cell-like structure in the potentially microbially formed pit, (**C**) potential mineralized structure, (**D**) nano-scale precipitates, (**F**) structure resembling a large stalked bacterium or fungal hyphae.

**Figure 3:**
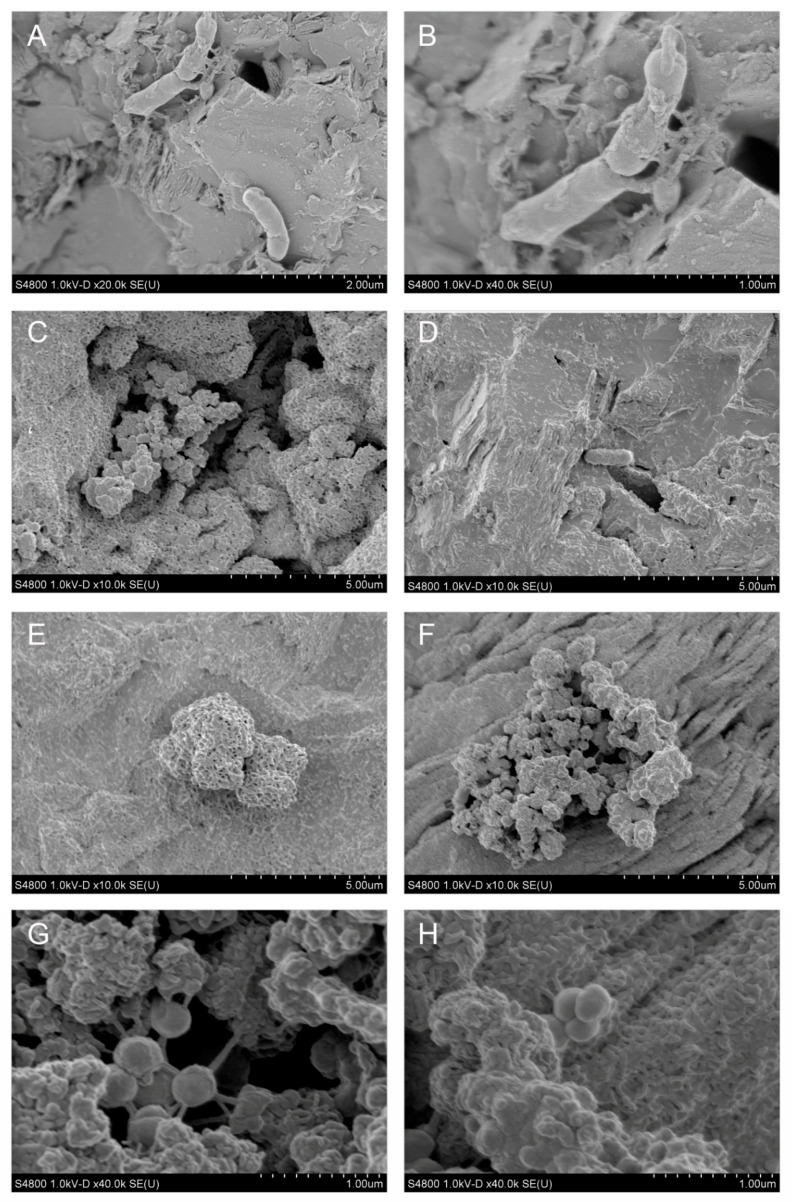
SEM analysis of the in situ incubated mica schist surfaces from the closed trap model (**A**,**B**) and open trap model (**C**–**H**) from the depth of 967 m. Each figure has a scale at the bottom for size reference (um = μm). (**A**) Potential bacterial cells with extracellular excretions, (**B**) close-up of (**A**), (**C**) biofilm-like structures on rock surface, (**D**) cell-like structure, (**E**) small spheroid structures, (**F**) consortia of assumed small-sized cells showing nanotube-like filamentous intercellular structures (**G**), and coccoid attached cell-like-clusters (**H**).

After 6 months of incubation, an initial biofilm of sparsely distributed microbial cells was detected on the mica schist slides. When examined with SEM, the biofilm consisted mainly of a single layer of microbial cells and cell-like aggregates attached on the mica schist surfaces (Figure 2 and Figure 3). In addition, areas with denser biofilm-like matrix with assumed EPS were detected (Figure 3C). The observed microbial cell morphology types included rods, cocci, and potentially small fungi or large stalked bacteria. Microbial cell sizes determined with SEM varied from 0.5 to over 4 µm. Microbial cells and their surroundings showed potential structures, enabling them to attach to surfaces including potential extracellular secretions (e.g., Figure 3A,B), microbially induced pits on rock surface (Figure 2B,E) and nano-scale precipitates (e.g., Figure 2D). In addition, we observed a cluster with potential nanobacteria-like structures attached to one another with nanowire-like or nanotube-like threads and small spheroid secretions or cell mass surrounding these structures (Figure 3F) [76,77,78].

### 3.4. Mineralogical Measurements with SEM

Mineralogy of the mica schist slides was investigated with SEM (EDS mode) both prior and after the 500 m depth in situ experiment (Figures S4 and S5). The main rock forming minerals detected were quartz, biotite, feldspar, and chlorite with accessory calcite, rutile, apatite, Fe-Mg-bearing garnet, and zircon. In addition to calcite, carbon was found in many other spots in the initial samples (Figure S4). Graphite is common in the Outokumpu metasediments [79] and some of the carbon detected with SEM could be morphologically identified as graphite. Some carbon spots could be refractory organic carbon compounds, as described by [80], or due to contamination of the SEM chamber, as carbon is commonly used to coat samples. However, carbon containing aggregates were generally larger (25–75 μm) after the samples had been exposed for six months to in situ conditions in the biofilm trap (Figure S5). After the experiment, carbon-rich spots were also found to contain chlorine, likely originating from the saline groundwater. In the samples derived after the experiment from the 500 m trap, fibrous or needle like Ti-rich particles, possibly rutile, were also found. Neither chemical nor visual changes in the main mineralogy of the samples were observed with SEM.

### 3.5. Microbial Communities

#### 3.5.1. Fungi

**Figure 4:**
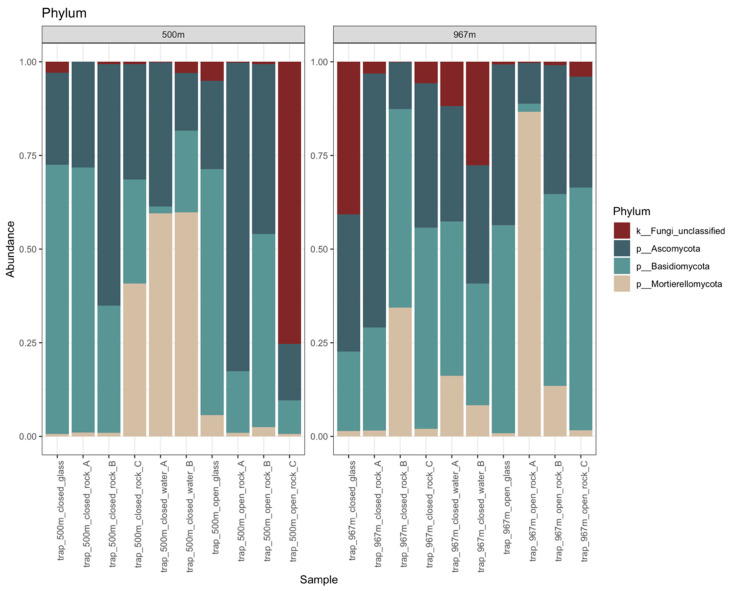
The relative abundance of fungal phyla detected at depths 500 and 967 m. In the legend on the right, k in front of the taxon name indicates identification to kingdom level, p indicates identification to phylum level.

The total number of fungal sequences was 28,868 and the number of detected fungal OTUs was 1832 (Table S2a). Library sizes varied from 689 to 4020 sequences/sample with a mean library size of 1949 sequences/sample (std.dev of 960) (Table S3a). The fungal communities showed high diversity. The detected three main phyla were Basidiomycota, Ascomycota, and Mortierellomycota, but 0–75% (with ~9% on average) of the sequence reads were only annotated as unclassified Fungi (Figure 4). Both the Basidiomycota and the Ascomycota phyla were divided to 9 different classes. In addition, both phyla hosted a variety of different fungal taxa that remained unclassified at class or order level, such as unclassified Tremellomycetes. In the negative control samples, the most abundant fungal OTUs were unclassified Nectriaceae, unclassified Trichosporon, and *Cutaneotrichoporon mucoides*. In total, 186 fungal OTUs present in the negative control samples were filtered from the data.

**Figure 5:**
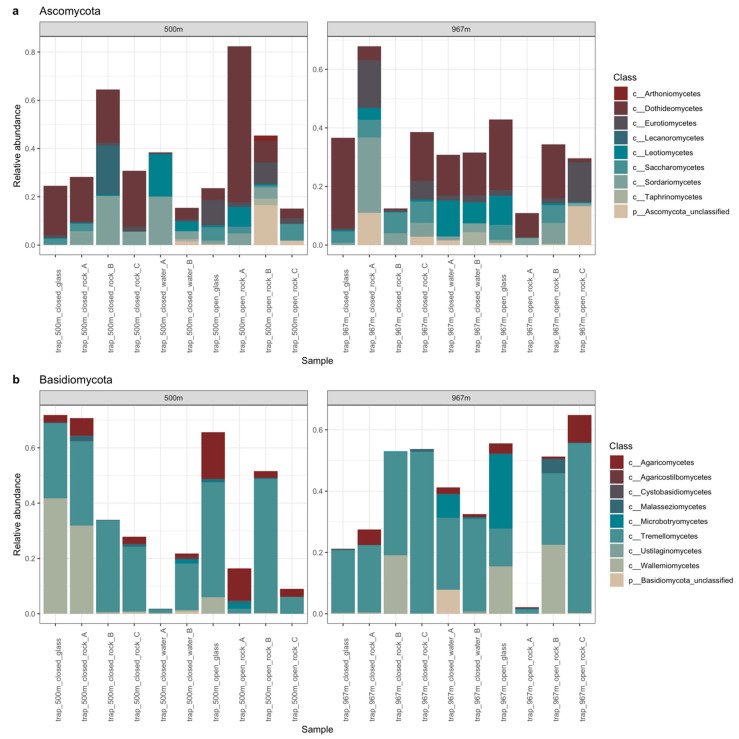
The relative abundance of basidiomycotal (**a**) and ascomycotal (**b**) classes at 500 and 967 m depths. In the legend on the right, c in front of the taxon name indicates identification to class level, p indicates identification to phylum level.

**Figure 6:**
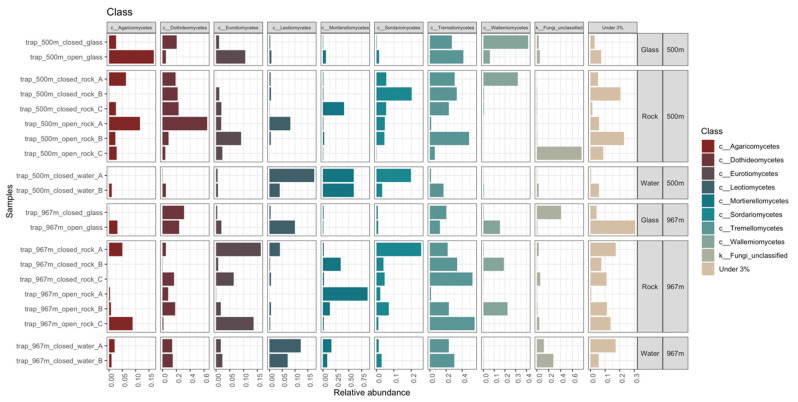
The fungal communities at the different sampling depths (500 or 967 m) and on different surfaces (glass, rock, water) presented on class level. Notice that in this figure, there is a class-wise relative abundance level on x-axis, compare samples within class, not across classes. Classes present at less than 3% relative abundance were merged as group “Under 3%”. In the legend on the right, c in front of the taxon name indicates identification to class level, k indicates identification to kingdom level.

The main identified fungal classes were the ascomycotal Dothideomycetes (17% on average) and the basidiomycotal Tremellomycetes (25%) in all sample types (Figure 5a,b, and Figure 6). In addition, the Mortierellomycota was present in all samples (17%). This class consisted only of the genus *Mortierella*, which had higher relative abundance on rock surfaces (16%) and in water (36%) than on glass surfaces (2%). Genus *Mortierella* was more prevalent in water samples at 500 m depth (60%) compared to the 967 m depth (12%), and more prevalent on mica schist at 967 m depth (23%) than at 500 m depth (8%). The glass surface samples contained mainly the Tremellomycetes (25%) and ascomycotal classes Dothideomycetes (20%) and Wallemiomycetes (16%) (Figure 6). Despite lower overall relative abundance, Dothideomycetes was more pronounced on mica schist surfaces (4.6%) than in water samples (1.3%). Furthermore, Saccharomycetes was more common on both mica schist (3.4%) and glass surfaces (4.2%) compared to the planktic state (0.1%). The relative abundance of Sordatiomycetes was higher in the closed biofilm traps (9% and 7% for 500 and 967 m, respectively) compared to the open trap (3% for both 500 and 967 m), which had unrestricted gas and fluid exchange with the surrounding deep subsurface. Similar observations were made for Wallemiomycetes, which made up 13% and 9% of the fungal communities in the 500 and 967 m open traps, but only 2% and 3% in the 500 and 967 m closed traps, respectively. Contrastingly, Agaricomycetes had higher relative abundance in the open traps (6%) than in the closed traps (2%), and the open 500 m trap showed higher proportion of Eurotiomycetes (6%) than did the closed 500 m trap (1%).

**Figure 7:**
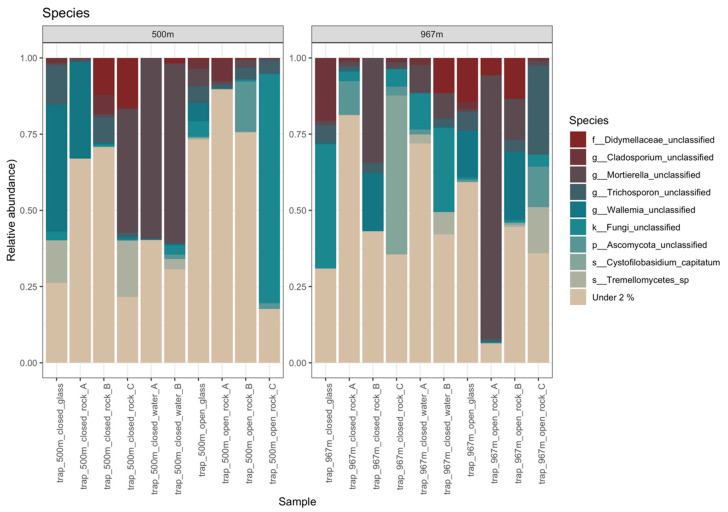
Fungal species in samples from 500 m (**left**) and 967 m (**right**). All the species present at less than 2% relative abundance were merged as group “Under 2%”. In the legend on the right, f, g, k, p, or s in front of the taxon name indicates identification to family, genus, kingdom, phylum, and species level, respectively.

The most common detected fungal group at species-level was unclassified *Mortierella* sp. (with ~17% on average) (Figure 7, Table S4). Most OTU groups remained without taxonomical classification at the species level, such as OTUs falling with unclassified Fungi (with ~9% on average), unclassified Mortierella (~17%), and unclassified Ascomycota (~3%). The most prevalent species with an even distribution across samples with available known reference species were *Vishniacozyma victoriae* (with ~2% on average) and *Vischniacozyma heimayensis* (~2%) and *Debaryomyces hansenii* (~2%). In addition, unclassified *Mortierella* (with ~16% on average), unclassified Fungi (~8%), unclassified *Wallemia* (~6%), unclassified *Trichosporon* (~5%), unclassified Didymellaceae (~4%), *Tremellomycetes* sp. (~3%), *Vishniacozyma heimaeyensis* (~3%), unclassified Aspergillus (~2%), and *Debaryomyces hansenii* (~2%) were commonly identified fungal types across several mica schist samples. However, many of the observed fungal OTUs had less than 1% prevalence in any sample.

#### 3.5.2. Bacteria

**Figure 8:**
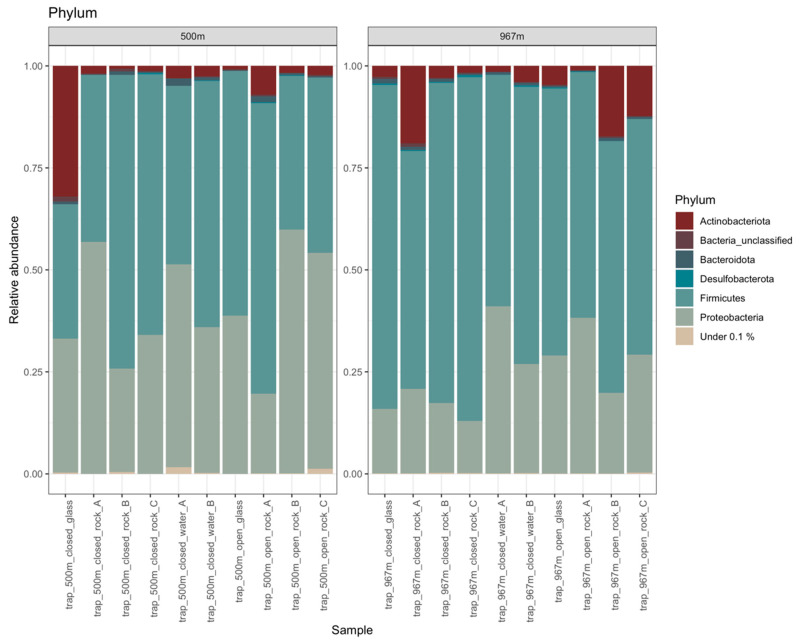
Relative abundance of bacterial phyla from sampling depths 500 and 967 m. All phyla present at less than 0.1% relative abundance are merged as group “Under 0.1%”.

**Figure 9:**
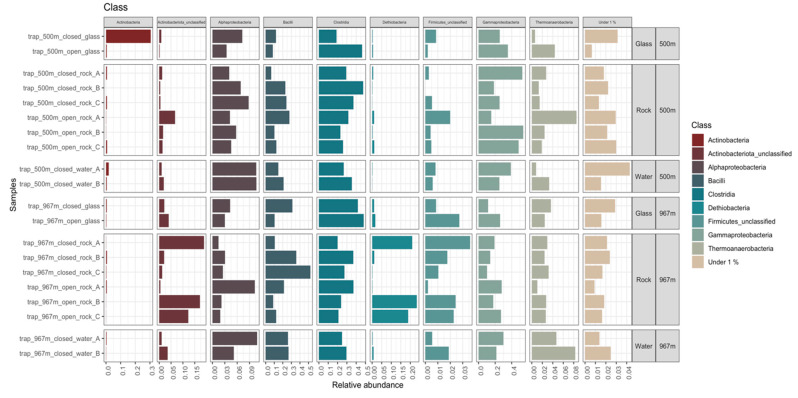
Bacterial communities from depths 500 and 967 m, and on different surfaces (glass, rock, water) presented at class level. All classes present at less than 1% relative abundance are merged as group “Under 1%”. Notice class-wise relative abundance and compare samples within class, not across classes.

A total of 27,749 bacterial 16S rRNA gene sequence reads were obtained, which were divided in 706 OTUs (Table S2b). The mean bacterial sequence counts per sample was 1387.5 (std.dev 541), with minimum counts of 453 and maximum counts of 2348 sequences per samples (Table S3b). The major phyla representing the bacterial communities were Firmicutes, Proteobacteria, and Actinobacteriota (Figure 8). On mica schist surfaces, the relative abundance of Firmicutes was 38–84% (with ~61% on average), Proteobacteria 13–60% (~32%), and Actinobacteriota 0.7–19% (~6%). The bacterial communities had high diversity with Bacteroidota, Desulfobacterota, Chloroflexi, Cyanobacteria, Patescibacteria, and unclassified Bacteria present as minor groups in many samples. The Firmicutes phylum was represented especially by *Erysipelothrichales*, *Peptostreptococcales-Tissierellales*, *Acholeplasmatales*, *Thermoanaerobacterales*, *Dethiobacterales*, *Desulfitobacteriales* and *Clostridiales* in most of the samples (Figure S3f). Dethiobacteria was more common in mica schist samples at 967 m than in other samples. The most prominent proteobacterial group was the Gammaproteobacteria. The mica schist samples from 500 m depth had a higher relative abundance of *Burkholderiales* (~35%) and *Xanthomonadales* (~0.43%) compared to other sample types (~30.6%, ~0.14%, respectively) (Figure S3g). The detected Actinobacteria affiliated mainly with unclassified Actinobacteria, which were more common on mica schist surfaces from 967 m depth (Figure S3a). In addition, most of the detected Cyanobacteria were found attached to rock surfaces, not planktic (Figure S3e). Bacterial classes detected in higher relative abundance on mica schist surfaces included Bacilli at both sampling depths, Desulfitobacteria and Gammaproteobacteria especially at 500 m depth and unclassified Actinobacteria, unclassified Firmicutes, and Dethiobacteria especially at 967 m depth (Figure 9). The most abundant contaminant bacterial OTUs were unclassified *Alicyclobacillaceae*, *Mycobacterium*, unclassified *Sphingomonadaceae*, and unclassified *Xanthobacteraceae.* In total, 48 bacterial OTUs were filtered from the data based on negative controls.

### 3.6. Statistical Analyses

**Figure 10:**
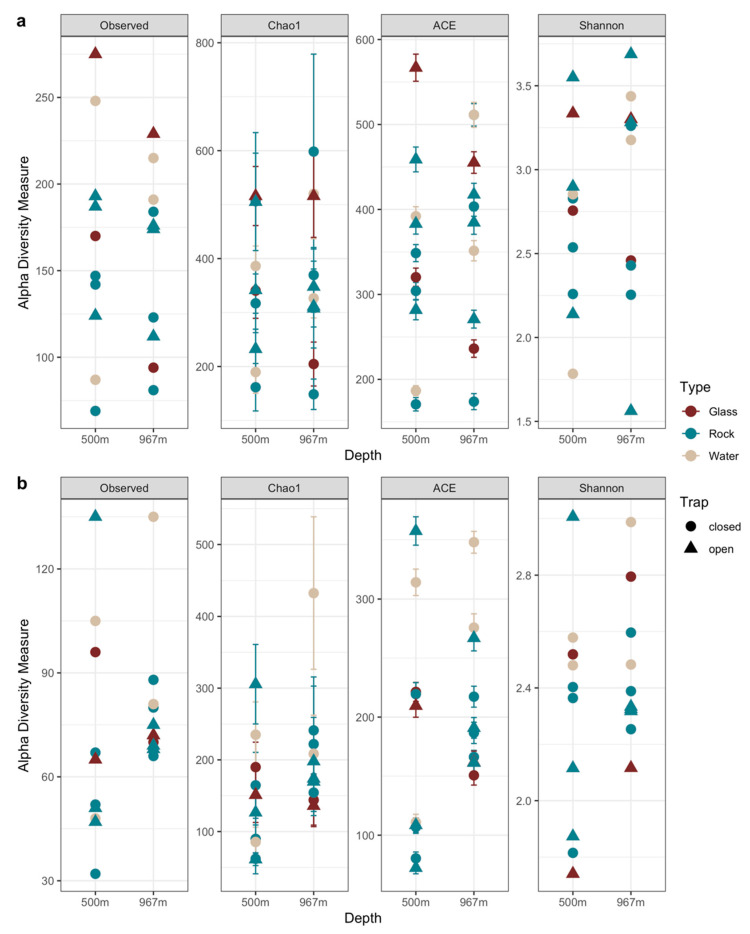
Alpha diversity measures of fungal (**a**) and bacterial (**b**) communities at different sampling depths. From the left, observed OTUs, Chao1 estimated number of OTUs, ACE estimated number of OTUs, Shannon’s diversity index *H*′. The error bars in the Chao1 and ACE richness graphs describe standard error (se), se.chao1, and se.ACE.

The number of observed species in the fungal communities varied between 69–275 and 81–221 in all different sample types from 500 and 967 m depths, respectively (Figure 10a, Table S2a). Shannon’s diversity index (*H*′) varied from 1.6 to 3.6 in all samples. The fungal communities identified from the open biofilm traps had higher *H*′ compared to the closed biofilm trap fungal communities (Figure 10a). The *H*’ for the water samples from the closed trap at 967 m was higher (3.2–3.4) compared to that of the 500 m water samples (1.8–2.9). However, all sample types and sampling depths contained samples with varying *H*’ values, indicating that the distribution of fungal cells across the matrix was not uniform. The estimated Chao1 richness varied between 149–598 (with standard error (se) ranging between ± 28–181) and abundance-based coverage estimate (ACE) between 171–567 (with se ± 8–16), respectively.

The lowest diversity index *H′* value of the bacterial communities was detected from the glass surface samples from the open trap model at both sampling depths, whereas water and glass samples from the closed traps had the highest *H*’ (Figure 10b, Table S2b). Overall, *H*’ of the bacterial communities varied from 3.3 to 5.0. The number of observed OTUs was highest in water and rock samples. For bacteria, the Chao1 estimate was 61–433 (with se ± 9–106), and ACE was 72–358 (with se ± 5–12). Both the Chao1 and ACE richness estimators predicted the OTU abundance higher compared to the total number of detected OTUs.

### 3.7. PCoA for Fungal and Bacterial Communities

**Figure 11:**
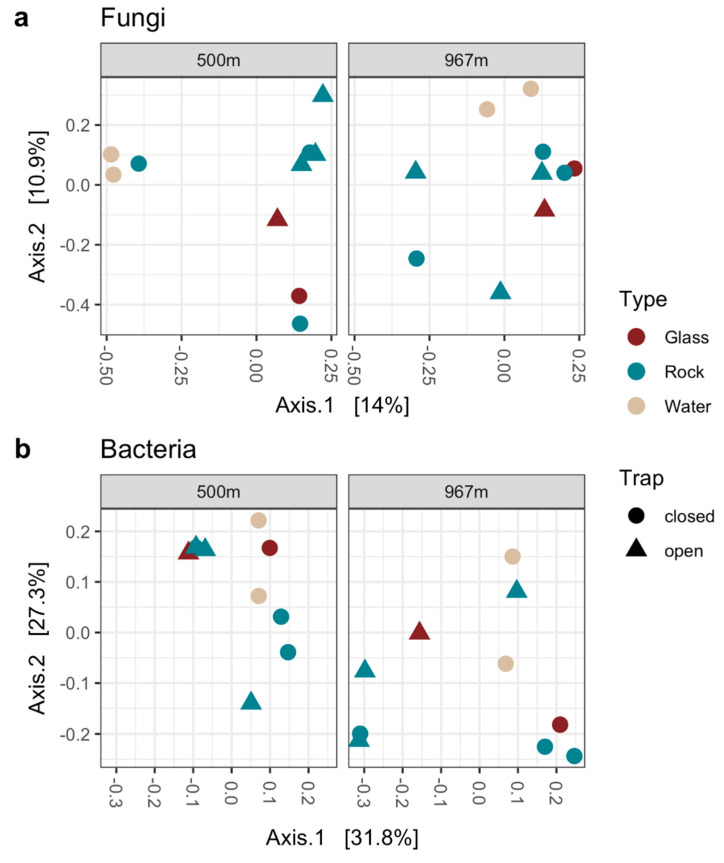
Fungal (**a**) and bacterial (**b**) community differences visualized with PCoA using the Bray–Curtis dissimilarity model. The PCoA was built from normalized count data. Axis 1 and 2 explains 14% and 10.9% of the variance for the fungal communities, respectively (**a**), and 31.8% and 27.3% of the variance for the bacterial communities, respectively (**b**).

**Figure 12:**
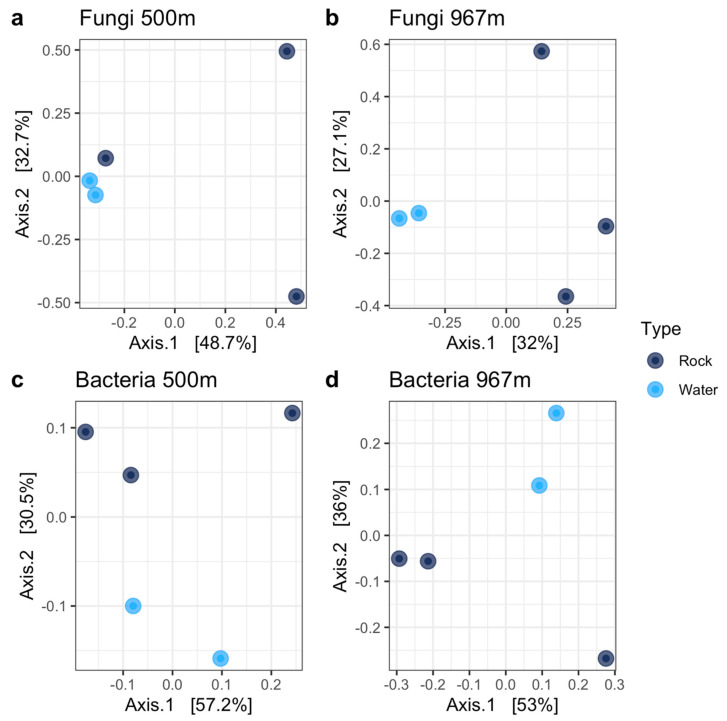
Comparison of microbial community composition in the closed traps were evaluated with PCoA. Fungal community composition in rock surface and water samples were analyzed from the depths of 500 m (**a**) and 967 m (**b**) and bacterial community composition at the depths of 500 m (**c**) and 967 m (**d**).

Similarities in the microbial community composition were analyzed with two types of PCoA, first the microbial communities between open and closed trap models were compared (Figure 11a–d), and then the closed trap planktic communities in water samples and sessile communities in mica schist surfaces were compared in detail (Figure 12a–d). The fungal communities in water samples differed from the fungal community composition present on rock or glass surfaces at both sampling depths (Figure 11a). The fungal communities of the open trap rock surface sample at 500 m grouped close together, indicating high similarities in community composition. In contrast, rock surface fungal communities at 967 m depth showed dissimilarities between samples. Glass surface fungal communities showed similarities with rock surface fungal communities arising from the same trap model (open, closed), while the depth showed no major effect on the fungal communities. The closed trap model showed dissimilarities between each rock surface fungal community. In addition, these mica schist fungal communities differed from the fungal community composition present in the water as only one rock surface sample group near to the water samples at 500 m depth. (Figure 12a,b). Fungal communities in the closed trap water group were very close together, indicating high similarities in community composition (Figure 12a,b). The bacterial community composition detected in samples derived from the different trap models (open, closed) showed dissimilarities at both sampling depths (Figure 11b). Bacterial communities on glass surfaces grouped closer to the water samples at 500 m depth, and closer to rock surface samples at 967 m depth. There were dissimilarities in bacterial community composition between water samples and rock surface samples from the closed trap (Figure 12c,d).

### 3.8. SPARCC-Correlations between OTUs

**Figure 13:**
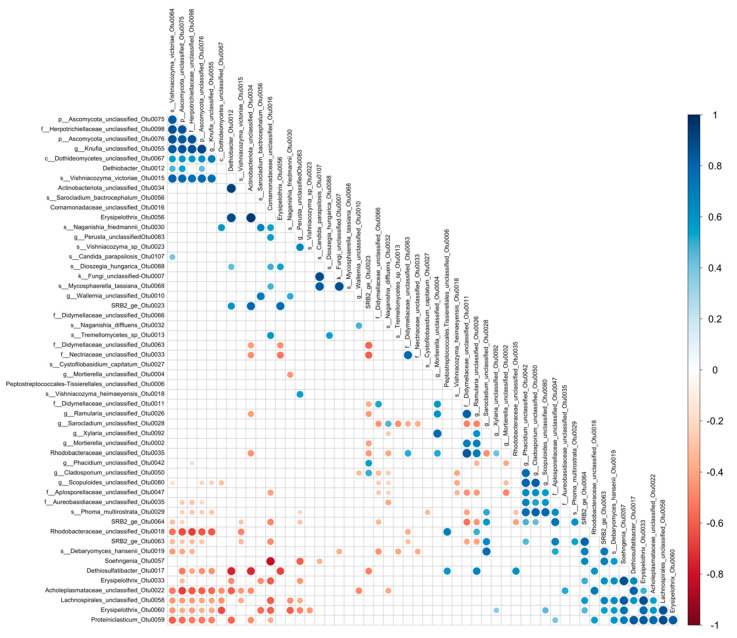
Cross-kingdom SPARCC correlations detected in the data. Only significant (*p* < 0.05) correlations between fungal and bacterial OTUs present in at least two samples (rock surface samples) with minimum 50 fungal sequence reads, or 20 bacterial sequence reads are shown. Blue color indicates positive correlations and red color negative correlations. The color scale on the right indicate the level of the correlation, with darker color for the strongest positive or negative correlations. In the taxon names, p, f, g, c, and s prefixes indicate identification to phylum, family, genus, class, or species level, respectively.

Significant OTU pair SPARCC-correlations were detected mainly between Fungi–Fungi or Bacteria–Bacteria, but also significant cross-kingdom correlations were observed (Figure 13, Table S6). For example, positive correlations were detected between sulfate reducing bacterial OTUs SRB2_OTU0063 or SRB2_OTU0064 with the fungal OTU *Debaryomyces hansenii*_OTU0019 (correlation > 0.6, pseudo *p*-value < 0.001). In addition, negative cross-kingdom correlations could be detected between bacterial OTU *Erysipelothrix*_OTU0060 and fungal OTU Dothideomycetes unclassified_Otu0067 (−0.6, *p* < 0.001). We detected significant correlations between the sulfate reducer SRB2_ge_OTU00063 and either fungal OTUs of unclassified *Sarocladium* (0.6, *p* < 0.01) or with unclassified *Aplosporellata* (0.7, *p* < 0.01). *Dethiobacter* OTU0012 showed significant correlation with unclassified Ascomycota fungi (0.53, *p* < 0.025), and with unclassified Actinobacteriota (0.9, *p* < 0.001) on mica schist surfaces. *Dethiosulfatibacter* showed also positive correlation with unclassified Ramularia fungi (0.7, 0.02) and with many bacteria, such as unclassified Acholeplasmataceae (0.8, *p* < 0.001). There was a significant negative correlation between *Dethiobacter* and *Dethiosulfatibacter* (−0.8, *p* < 0.001).

## 4. Discussion

Microbial life in the deep oligotrophic subsurface is considered to be slow and energy conserving [81,82]. However, synergistic relationships where one metabolic route feeds another, i.e., community-wide networks, may maintain a considerably active and diverse deep biosphere [83]. Fungal communities have been shown to actively transcribe their ribosomal genes in deep oligotrophic groundwater [35,42]. In this oligotrophic environment, microbial cells may gain several advantages from a sessile lifestyle on mineral surfaces, including resistance to environmental factors and possibility of cell-to-cell interaction [84]. To our knowledge, our study is the first to describe fungal ecology of the rock-surface attaching fungal communities in deep crystalline bedrock by applying in situ incubation, SEM and high-throughput amplicon sequencing. We exposed mica schist surfaces to in situ conditions for a 6 month period. Our observations suggest that the attachment rate of microbes on rock surfaces in Outokumpu deep bedrock in in situ conditions is a slow process dominated by fungi and bacteria.

Nevertheless, even over the short 6 month in situ incubation period, the diversity of the fungal and bacterial communities attached to the mica schist was surprisingly high. Our results are in contrast with reports on biofilms formed over longer time periods. For example, in a 10 year in situ incubation established in a deep continental drill hole between packers, the observed microbial community composition of the studied teflon surface biofilms were very uniform compared to the diversity observed in the water column [32]. In addition, the surrounding main rock type affected the formed biofilm community composition [32], which could indicate that natural rock surfaces may support more diverse communities compared to artificial teflon surfaces. Our study showed that a 6 month incubation period was not sufficient to form a uniform and evenly distributed biofilm on the mica schist in the present environment (e.g., Figure 12a–d), but during the establishment of the biofilm the diversity of the primary attached microorganisms is high. SEM visualization also showed that attached microbial communities varied from individual attached cells to more developed biofilm-like structures. Other studies have shown deep biofilms to host a great microbial diversity and also potential for complex inter-species interaction [12,27,85].

There is very limited information on continental, anoxic deep subsurface fungal community composition on rock surfaces. Our results showing fungal and bacterial dominance of deep rock surface communities are consistent with findings of Schäfer et al. (2015) that were able to amplify both bacterial and fungal DNA from granitic Äspö drill cores [25]. Another study reported the fungal phyla Ascomycota, Basidiomycota, and Cryptomycota from terrestrial deep sedimentary rock surfaces (depths of 250 and 270 m) in Horonabe, Japan [86]. This is in accordance with our study, where Ascomycota and Basidiomycota were the most prominent fungal phyla detected. However, we also found Mortierellomycota in the biofilms and a considerable portion of the fungal community that remained as unclassified Fungi. The attached fungal communities were highly diverse in the Outokumpu deep bedrock at both sampling depths of 500 and 967 m. Deep biosphere fungi appeared to have the ability to occupy and attach to different surfaces at in situ conditions within months. In our present study, the most prominent fungal genera detected on Outokumpu mica schist surfaces were *Mortierella* sp., *Vishniacozyma* sp., *Cladosporium* sp., *Debaryomyces* sp., *Cystofilobasidium* sp., *Naganishia* sp., and *Trichosporon* sp. Furthermore, our mica schist samples hosted genera of known epi—and endolithic rock inhabiting fungal species, such as *Aspergillus* sp., *Botrytis* sp., *Hormonema* sp., and *Verticillium* sp. [22]. We also observed unclassified *Pleosporales* and *Cladosporium* sp., genera that have previously been linked to oxidation of iron and manganese [22], on mica schist surfaces from both sampling depths. *Mortierella* sp. have been found from different hostile environments including hydrocarbon contaminated soils and heavy metal rich underground iron ore mines [87,88]. They have been shown to produce nitrous oxide (N_2_O), and host *Burkholderia*-related endosymbiotic bacteria [89]. Various types of *Burkholderia* are common in Outokumpu groundwater, especially in the deeper part of the bedrock [50,58]. Both fungi and *Burkholderia* could have an advantage due to endosymbiotic relationships, which could help them to adapt to deep subsurface conditions. *Cryptococcus*-type yeasts represent typical deep biosphere fungi, which have also been isolated from the deep groundwater in Äspö, Sweden [33]. Ekendahl et al. (2003) showed that these deep subsurface isolates of *Cryptococcus* yeasts produced thread-like exopolymers. Ability to produce this kind of excretions allows yeast cells to attach to mineral surfaces. The class Tremellomycetes containing the genus *Cryptococcus* has recently been reorganized and the *Cryptococcus* genus was split into new genera [90]. The newly emerged genera include *Vishniacozyma* and *Naganishia*, which were abundantly found in our rock samples. Interestingly, many of the fungal genera detected in our samples, especially yeasts, are found in harsh, dry conditions of the Antarctica or from Antarctic lakes [91,92]. Furthermore, the *Naganishia* genus is widespread globally and found from extreme environments, such as the Atacama Desert, and high elevated ecosystems exposed to drought and low nutrient availability [93]. *Debaryomyces* was another abundant halotolerant yeast genus on our mica schist surfaces, and it has been found in various saline environments including ocean water and salterns [94]. In summary, many of the fungal taxa that we detected in our deep in situ incubated rock surfaces have been shown to thrive or tolerate the extreme living conditions elsewhere.

Observed planktic cell counts within the closed biofilm traps were higher than the general cell counts in the fluids from these fracture zones [50]. We could see that bacterial community composition on mica schist surface differed from the bacterial communities in the water samples. Similarly, bacterial planktic and sessile drill core biofilm communities were distinctively different both in Äspö and SURF deep subsurface facilities [26,30]. Main bacterial phyla on our mica schist surfaces belonged to Firmicutes and Proteobacteria. Same bacterial phyla dominated drill core rock samples from SURF, and also fluid samples from other compared terrestrial deep subsurface sites [30]. Our dataset shows that known thiosulfate-reducing bacteria, such as the firmicutes *Dethiobacter* and *Dethiosulfatibacter*, were especially frequent on mica schist surfaces [95,96]. Furthermore, sulfate reducers (e.g., SRB2) and elemental sulfur utilizers (e.g., genera of the Desulfuromonadaceae) were found both on rock surfaces and in planktic state. SRB have been shown to make up a large portion of the total bacterial community at 500 m depth in Outokumpu fracture fluids and a smaller part of the bacterial community of the 967 m fracture zone [50].

Microbial metabolism modifies surrounding environment through modification of available substrates e.g., preferential use of lighter isotopes leads to accumulation of heavier isotopes in the residue [97]. Drake et al. (2017) visualized partially mineralized fungal structures as part of biofilms in crystalline bedrock drill cores [17]. Furthermore, they suggested rock surface biofilm Fungi–SRB interaction as they detected ^34^S depleted sulfur isotope signatures in pyrite crystals on hyphal structures thus supporting potential SRB activity [17,98]. In their conceptual model for the interactions, the SRB provide fungi with organic carbon and in turn hydrogen formed by the fungal metabolism is consumed by SRB [17]. In our study, we observed cross-kingdom correlations between fungi and bacterial groups linked to the utilization of sulfur and thiosulfate as electron acceptors on mica schist surfaces. This could indicate a potential for mutualistic interaction between fungi and bacteria, where these bacterial groups could gain advantage of the fungal leaching properties in harvesting the required electron acceptors, such as sulfur. In our study, SRB, e.g., Thermoanaerobacterales group SRB2, showed significant correlations with various fungi e.g., *Debaryomyces hansenii*. Previously, Ivarsson et al. (2016) suggested that deep biosphere fungi could produce part of the available hydrogen in the deep subsurface [99]. This fungal-produced hydrogen could be oxidized by mutualistic bacterial groups, such as *Dethiobacter* detected in this study. The type species of *Dethiobacter* was isolated from a soda lake and has been linked to the oxidation of hydrogen with thiosulfate, polysulfides, or sulfur as electron acceptors [89]. Besides providing hydrogen, fungi could benefit sulfur cycling bacteria in other ways and/or potentially participate in the deep sulfur cycle themselves. For example, *D. hansenii* has shown potential for production of various volatile organic sulfur compounds in culture, such as methanethiol, dimethylsulfide, dimethyldisulfide, dimethyltrisulfide, and methylthioacetate [100]. Similarly, basidiomycetous yeasts, such as *Cryptococcus* sp., have been reported to produce volatile organic sulfur compounds [101].

At surface conditions, rock-inhabiting fungi typically produce various organic acids (e.g., acetic, formic, fumaric, lactic, oxalic) in order to leach the rock surface to acquire their trace elements [22]. In deep biosphere, these acids could, in addition to leaching minerals from surrounding rock, help sustain diverse heterotrophic microbial communities as such in this very oligotrophic environment. For example, acetate is a carbon source for the microorganisms e.g., the chemolithotrophic *Dethiobacter* [96]. Similarly, the Outokumpu deep subsurface microbial communities have been shown to use acetate and benefit from acetate during enrichment [58,61]. Here, we showed that *Dethiobacter* attached to mica schist surfaces especially at 967 m depth. During the incubation, we also observed a major decrease in pH from 9.6 and 9.9 to 6.4 and 6.7 at 500 and 967 m, respectively, in the closed model traps (Table 2). In addition, the levels of various elements (e.g., Fe, P, S, Cu, Zn, As, and Ni) increased in the closed trap fluids, which could be due to microbial leaching of the mica schist in the traps. The elements fit well to the elemental composition of Outokumpu’s mica schist [53]. Further affirmation for microbial leaching of minerals was acquired by visual analysis using SEM where potential microbial erosion pits on mica schist surface were detected (Figure 2b,e). Stable isotope analysis of water (H and O) in the closed traps also showed depletion of ^2^H and enrichment of ^18^O during the incubation (Table S1). In comparison, there were no detectable change in these ratios in the water in the PA-tubings above the traps. These changes in traps could indicate reactions with the mica schist, metal-water interaction, or microbiological processes. Based on the direction of the change, evaporation of water and hydration of silicates can be ruled out [56,102,103]. Opposite to what we observed, biosynthesis of hydrogen containing molecules typically leads to enrichment of ^2^H in the remaining water [97]. Isotope fractionation of oxygen in water during the corrosion of iron metal is poorly understood, especially at low temperature [104]. Isotope fractionation in reactions producing molecular hydrogen, such as oxidation of iron minerals, generally lead to extreme ^2^H depletion in the H_2_ gas (e.g., Lin et al. (2005) [105]), and thus cannot account for the observed ^2^H depletion in water. However, the change is congruent with leachate of fluid inclusions (metamorphic fluids) from the mica schist plates and crushed rock. Whether the detected change is a consequence of a microbiological leaching remains unresolved.

Deep subsurface fungi have thus far been almost neglected in deep life studies, but despite their apparent low abundance, they may play a significant role in the initiation of biofilm formation in the deep subsurface. They may be the key microorganisms to recycle both senescent biofilms and ancient organic carbon and mineral nutrients from rock. Our methods provide the possibility for long-term in situ incubation, which may reveal how the biofilm develops over time, and that the deep subsurface fungi should not be left out of the equation in the future.

## 5. Conclusions

Diverse fungal populations colonized rock surfaces during 6 month in situ incubation in deep continental bedrock in Outokumpu, Finland. Deep groundwater was shown to support diverse fungal communities in the in situ pressure in Outokumpu deep biosphere at the depths of 500 and 967 m. Fungal communities on mica schist surfaces differed from planktic fungal communities. Our study showed potential for mutualistic lifestyle between fungi and bacteria during the first steps of biofilm formation on rock surfaces in deep biosphere. The custom designed in situ biofilm trap was applicable in capturing the initiation of biofilm formation without the pressure drop during sampling, allowing in situ gas exchange with open trap model and enabling the collection of both fluid and biofilm incubation samples.

## Figures and Tables

**Table 1:** Sample types collected from incubated biofilm trap or the connected tube above. A similar set of samples was collected from the trap from each sampling depth (500 or 967 m).

Depth	Trap	Type	Analysis	Samples
500/967 m	Open	Rock	DNA	crushed mica schist × 3
			SEM	mica schist slides × 3 fixed with glutaraldehyde
		Glass	DNA	halved glass slides × 2
	Closed	Water	DNA	2 × Sterivex filter (100 mL/500 m, 50 mL/967 m)
			Microscopy	2 × 60 mL in a headspace bottle
			Cations	100 mL 0.22 µm filtrated + 0.5 mL HNO_3_
			Anions	150 mL fluid samples
			Isotopes	60 mL fluid samples
		Rock	DNA	crushed mica schist × 3
			SEM	mica schist slides × 3 fixed with glutaraldehyde
		Glass	DNA	halved glass slides × 2

**Table 2:** Geochemistry of the original situation in fracture fluids and in the closed biofilm trap after 6 months incubation. Samples 1 and 2 represent replicates.

Sample	EC	pH	Alkalinity	Ca	Cl	K	Na	Mg	Fe	P	S
	mSm^−1^ (25 °C)		mmol L^−1^	mg L^−1^	mg L^−1^	mg L^−1^	mg L^−1^	mg L^−1^	mg L^−1^	mg L^−1^	mg L^−1^
**Original**											
500 m_1	1940	9.6	0.2	2280	8150	17.6	1820	12	<0.03	<0.05	3.47
500 m_2	1940	9.6	0.2	2120	8690	17.4	1650	12	0.19	<0.05	3.69
967 m_1	2030	9.9	0.23	2420	8280	15.1	1900	5.94	<0.03	<0.05	2.08
967 m_2	2030	9.9	0.29	2270	8210	15.3	1830	5.97	<0.03	<0.05	2.18
**Incubated**											
500 m	1800	6.4	0.63	2350	7610	19.25	1865	11.05	0.5	0.44	4.23
967 m	1840	6.7	0.73	2490	7510	17.6	1920	5.09	0.21	0.52	7.71

## Data Availability

Sequence data is available in a publicly accessible repository European Nucleotide Archive (ENA) under project PRJEB40820 and accession numbers ERS5208985-ERS5209024. Additional data and files are available in supplementary material of this publication.

## Supplementary Materials

The following are available online at https://www.mdpi.com/2076-2607/9/1/64/s1, Figure S1: The in situ biofilm trap, Figure S2a–e: Inner parts of the in situ biofilm trap, Figure S3: Online monitoring parameters for pH, electrical conductivity, dissolved O_2_ and redox potential (Eh vs. SHE) for water pumped through the traps from the depth of 500 m (right) and the depth of 967 m (left) before initiation of the incubation period. Figure S4: Examples of back scattered electron (BSE) images, chemical composition in weight-%, and interpreted mineralogy of mica schist plates from 500 m depth before the biofilm trap experiment, Figure S5: Examples of back scattered electron (BSE) images, chemical composition in weight-%, and interpreted mineralogy of mica schist plates from 500 m depth after the biofilm trap experiment. Figure S6a–g: Additional taxonomy figures for Bacteria, Table S1: Additional geochemistry of the original situation in fracture fluids and in the biofilm trap after 6 months incubation. Table S2 (a,b): Alphadiversity measures of fungal (a) and bacterial (b) communities, Table S3 (a,b): Library sizes for fungal (a) and bacterial (b) communities, Table S4: OTU table for in situ biofilm trap samples, Fungal taxonomy, Table S5: OTU table for in situ biofilm trap samples, Bacterial taxonomy, Table S6 (a–c): SPARCC-correlations between rock surface microbial communities for (a) Bacteria—Fungi, (b) Fungi—Fungi, (c) Bacteria—Bacteria.

## References

[1] Magnabosco C., Lin L.-H., Dong H., Bomberg M., Ghiorse W., Stan-Lotter H., Pedersen K., Kieft T.L., Van Heerden E., Onstott T.C. (2018). The biomass and biodiversity of the continental subsurface. Nat. Geosci..

[2] McMahon S., Parnell J. (2014). Weighing the deep continental biosphere. FEMS Microbiol. Ecol..

[3] Coleine C., Stajich J.E., Zucconi L., Onofri S., Pombubpa N., Egidi E., Franks A., Buzzini P., Selbmann L. (2018). Antarctic Cryptoendolithic Fungal Communities Are Highly Adapted and Dominated by Lecanoromycetes and Dothideomycetes. Front. Microbiol..

[4] Selbmann L., Zucconi L., Isola D., Onofri S. (2015). Rock black fungi: Excellence in the extremes, from the Antarctic to space. Curr. Genet..

[5] Selbmann L., Grube M., Onofri S., Isola D., Zucconi L. (2013). Antarctic Epilithic Lichens as Niches for Black Meristematic Fungi. Biology.

[6] Ivarsson M., Bengtson S., Drake H., Francis W. (2017). Fungi in Deep Subsurface Environments. Adv. Appl. Microbiol..

[7] Pedersen K., Arlinger J., Ekendahl S., Hallbeck L. (1996). 16S rRNA gene diversity of attached and unattached bacteria in boreholes along the access tunnel to the Äspö hard rock laboratory, Sweden. FEMS Microbiol. Ecol..

[8] Ekendahl S., Arlinger J., Stahl F., Pedersen K. (1994). Characterization of attached bacterial populations in deep granitic groundwater from the Stripa research mine by 16S rRNA gene sequencing and scanning electron microscopy. Microbiology.

[9] Ekendahl S., Pedersen K. (1994). Carbon transformations by attached bacterial populations in granitic groundwater from deep crystalline bed-rock of the Stripa research mine. Microbiology.

[10] Wanger G., Southam G., Onstott T.C. (2006). Structural and Chemical Characterization of a Natural Fracture Surface from 2.8 Kilometers Below Land Surface: Biofilms in the Deep Subsurface. Geomicrobiol. J..

[11] Borgonie G., Magnabosco C., Garcia-Moyano A., Linage-Alvarez B., Ojo A.O., Freese L.B., Van Jaarsveld C., Van Rooyen C., Kuloyo O., Cason E.D. (2019). New ecosystems in the deep subsurface follow the flow of water driven by geological activity. Sci. Rep..

[12] Jägevall S., Rabe L., Pedersen K. (2011). Abundance and Diversity of Biofilms in Natural and Artificial Aquifers of the Äspö Hard Rock Laboratory, Sweden. Microb. Ecol..

[13] Pedersen K. (2012). Subterranean microbial populations metabolize hydrogen and acetate under in situ conditions in granitic groundwater at 450 m depth in the Äspö Hard Rock Laboratory, Sweden. FEMS Microbiol. Ecol..

[14] Dutta A., Gupta S.D., Gupta A., Sarkar J., Roy S., Mukherjee A., Sar P. (2018). Exploration of deep terrestrial subsurface microbiome in Late Cretaceous Deccan traps and underlying Archean basement, India. Sci. Rep..

[15] Smith A.R., Kieft B., Mueller R., Fisk M.R., Mason O.U., Popa R., Colwell F.S. (2019). Carbon fixation and energy metabolisms of a subseafloor olivine biofilm. ISME J..

[16] Ramírez G.A., Garber A.I., Lecoeuvre A., D’Angelo T., Wheat C.G., Orcutt B.N. (2019). Ecology of Subseafloor Crustal Biofilms. Front. Microbiol..

[17] Drake H., Ivarsson M., Bengtson S., Heim C., Siljeström S., Whitehouse M.J., Broman C., Belivanova V., Åström M.E. (2017). Anaerobic consortia of fungi and sulfate reducing bacteria in deep granite fractures. Nat. Commun..

[18] Drake H., Ivarsson M., Tillberg M., Whitehouse M.J., Kooijman E. (2018). Ancient Microbial Activity in Deep Hydraulically Conductive Fracture Zones within the Forsmark Target Area for Geological Nuclear Waste Disposal, Sweden. Geosciences.

[19] Reitner J., Schumann G., Pedersen K., Gadd G.M. (2006). Fungi in subterranean environments. Fungi in Biogeochemical Cycles.

[20] Gerrits R., Pokharel R., Breitenbach R., Radnik J., Feldmann I., Schuessler J.A., Von Blanckenburg F., Gorbushina A.A., Schott J. (2020). How the rock-inhabiting fungus K. petricola A95 enhances olivine dissolution through attachment. Geochim. Cosmochim. Acta.

[21] Gadd G.M. (2007). Geomycology: Biogeochemical transformations of rocks, minerals, metals and radionuclides by fungi, bioweathering and bioremediation. Mycol. Res..

[22] Sterflinger K. (2000). Fungi as Geologic Agents. Geomicrobiol. J..

[23] Casar C.P., Kruger B.R., Flynn T.M., Masterson A.L., Momper L.M., Osburn M.R. (2020). Mineral-hosted biofilm communities in the continental deep subsurface, Deep Mine Microbial Observatory, SD, USA. Geobiology.

[24] Leefmann T., Heim C., Lausmaa J., Sjövall P., Ionescu D., Reitner J., Thiel V. (2015). An Imaging Mass Spectrometry Study on the Formation of Conditioning Films and Biofilms in the Subsurface (Äspö Hard Rock Laboratory, SE Sweden). Geomicrobiol. J..

[25] Schäfer N., Schmidt B.C., Quéric N.V., Röring B., Reitner J. (2015). Organic Compounds and Conditioning Films Within Deep Rock Fractures of the Äspö Hard Rock Laboratory, Sweden. Geomicrobiol. J..

[26] Wu X., Pedersen K., Edlund J., Eriksson L., Åström M., Andersson A.F., Bertilsson S., Dopson M. (2017). Potential for hydrogen-oxidizing chemolithoautotrophic and diazotrophic populations to initiate biofilm formation in oligotrophic, deep terrestrial subsurface waters. Microbiome.

[27] Escudero C., Vera M., Oggerin M., Amils R. (2018). Active microbial biofilms in deep poor porous continental subsurface rocks. Sci. Rep..

[28] Wu X., Holmfeldt K., Hubalek V., Lundin D., Åström M., Bertilsson S., Dopson M. (2016). Microbial metagenomes from three aquifers in the Fennoscandian shield terrestrial deep biosphere reveal metabolic partitioning among populations. ISME J..

[29] Jangir Y., Karbelkar A.A., Beedle N.M., Zinke L.A., Wanger G., Anderson C.M., Reese B.K., Amend J.P., El-Naggar M.Y. (2019). In situ Electrochemical Studies of the Terrestrial Deep Subsurface Biosphere at the Sanford Underground Research Facility, South Dakota, USA. Front. Energy Res..

[30] Momper L., Reese B.K., Zinke L., Wanger G., Osburn M.R., Moser D., Amend J.P. (2017). Major phylum-level differences between porefluid and host rock bacterial communities in the terrestrial deep subsurface. Environ. Microbiol. Rep..

[31] Leandro T., Rodriguez N., Rojas P., Sanz J.L., Da Costa M.S., Amils R. (2018). Study of methanogenic enrichment cultures of rock cores from the deep subsurface of the Iberian Pyritic Belt. Heliyon.

[32] Amano Y., Iwatsuki T., Naganuma T. (2017). Characteristics of Naturally Grown Biofilms in Deep Groundwaters and Their Heavy Metal Sorption Property in a Deep Subsurface Environment. Geomicrobiol. J..

[33] Ekendahl S., O’Neill A.H., Thomsson E., Pedersen K. (2003). Characterisation of Yeasts Isolated from Deep Igneous Rock Aquifers of the Fennoscandian Shield. Microb. Ecol..

[34] Purkamo L., Kietäväinen R., Miettinen H., Sohlberg E., Kukkonen I., Itävaara M., Bomberg M. (2018). Diversity and functionality of archaeal, bacterial and fungal communities in deep Archaean bedrock groundwater. FEMS Microbiol. Ecol..

[35] Bomberg M., Raulio M., Jylhä S., Mueller C.W., Höschen C., Rajala P., Purkamo L., Kietäväinen R., Ahonen L., Itävaara M. (2017). CO2 and carbonate as substrate for the activation of the microbial community in 180 m deep bedrock fracture fluid of Outokumpu Deep Drill Hole, Finland. AIMS Microbiol..

[36] Orsi W., Biddle J.F., Edgcomb V. (2013). Deep Sequencing of Subseafloor Eukaryotic rRNA Reveals Active Fungi across Marine Subsurface Provinces. PLoS ONE.

[37] Purkamo L., Kietäväinen R., Nuppunen-Puputti M., Bomberg M., Cousins C. (2020). Ultradeep Microbial Communities at 4.4 km within Crystalline Bedrock: Implications for Habitability in a Planetary Context. Life.

[38] Miettinen H., Kietäväinen R., Sohlberg E., Numminen M., Ahonen L., Itävaara M. (2015). Microbiome composition and geochemical characteristics of deep subsurface high-pressure environment, Pyhäsalmi mine Finland. Front. Microbiol..

[39] Sinclair J.L., Ghiorse W.C. (1989). Distribution of aerobic bacteria, protozoa, algae, and fungi in deep subsurface sediments. Geomicrobiol. J..

[40] Fliermans C.B. (1989). Microbial Life in the Terrestrial Subsurface of Southeastern Coastal Plain Sediments. Hazard. Waste Hazard. Mater..

[41] Bomberg M., Mäkinen J., Salo M., Kinnunen P. (2019). High Diversity in Iron Cycling Microbial Communities in Acidic, Iron-Rich Water of the Pyhäsalmi Mine, Finland. Geofluids.

[42] Sohlberg E., Bomberg M., Miettinen H., Nyyssãnen M., Salavirta H., Vikman M., Itãvaara M. (2015). Revealing the unexplored fungal communities in deep groundwater of crystalline bedrock fracture zones in Olkiluoto, Finland. Front. Microbiol..

[43] Borgonie G., Linage-Alvarez B., Ojo A.O., Mundle S.O.C., Freese L.B., Van Rooyen C., Kuloyo O., Albertyn J., Pohl C., Cason E.D. (2015). Eukaryotic opportunists dominate the deep-subsurface biosphere in South Africa. Nat. Commun..

[44] Pedersen K. (1987). Preliminary Investigations of Deep Ground Water Microbiology in Swedish Granitic Rock.

[45] Boddy L., Watkinson S.C., Boddy L., Money N.P. (2015). Interactions Between Fungi and Other Microbes. The Fungi.

[46] Kukkonen I.T., Rath V., Kivekäs L., Šafanda J., Čermak V. (2011). Geothermal studies of the Outokumpu Deep Drill Hole, Finland: Vertical variation in heat flow and palaeoclimatic implications. Phys. Earth Planet. Inter..

[47] Ahonen L., Kietäväinen R., Kortelainen N., Kukkonen I.T., Pullinen A., Toppi T., Bomberg M., Itävaara M., Nousiainen A., Nyyssönen M., Kukkonen I.T. (2011). Hydrogeological characteristics of the Outokumpu Deep Drill Hole. Geological Survey of Finland, Special Paper 51. Outokumpu Deep Drilling Project 2003–2010.

[48] Itävaara M., Nyyssönen M., Kapanen A., Nousiainen A., Ahonen L., Kukkonen I. (2011). Characterization of bacterial diversity to a depth of 1500 m in the Outokumpu deep borehole, Fennoscandian Shield. FEMS Microbiol. Ecol..

[49] Nyyssönen M., Hultman J., Ahonen L., Kukkonen I., Paulin L., Laine P., Itävaara M., Auvinen P. (2014). Taxonomically and functionally diverse microbial communities in deep crystalline rocks of the Fennoscandian shield. ISME J..

[50] Purkamo L., Bomberg M., Nyyssönen M., Kukkonen I., Ahonen L., Kietäväinen R., Itävaara M. (2013). Dissecting the deep biosphere: Retrieving authentic microbial communities from packer-isolated deep crystalline bedrock fracture zones. FEMS Microbiol. Ecol..

[51] Rajala P., Bomberg M., Kietäväinen R., Kukkonen I., Ahonen L., Nyyssönen M., Itävaara M. (2015). Rapid Reactivation of Deep Subsurface Microbes in the Presence of C-1 Compounds. Microorganisms.

[52] Hölttä P., Karttunen P., Kukkonen I.T. (2011). Metamorphism as a function of depth in metasedimentary rocks of the Outokumpu Deep Drill Hole. Geological Survey of Finland, Special Paper 51. Outokumpu Deep Drilling Project 2003–2010.

[53] Västi K., Kukkonen I.T. (2011). Petrology of the drill hole R2500 at Outokumpu, Eastern Finland—The deepest drill hole ever drilled in Finland. Geological Survey of Finland, Special Paper 51. Outokumpu Deep Drilling Project 2003–2010.

[54] Sharma P., Tsang C.-F., Kukkonen I.T., Niemi A. (2015). Analysis of 6-year fluid electric conductivity logs to evaluate the hydraulic structure of the deep drill hole at Outokumpu, Finland. Int. J. Earth Sci..

[55] Kietäväinen R. (2017). Deep Groundwater Evolution at Outokumpu, Eastern Finland: From Meteoric Water to Saline Gas-Rich Fluid. Special Publication 97 (Academic Dissertation).

[56] Kietäväinen R., Ahonen L., Kukkonen I.T., Hendriksson N., Nyyssönen M., Itävaara M. (2013). Characterisation and isotopic evolution of saline waters of the Outokumpu Deep Drill Hole, Finland—Implications for water origin and deep terrestrial biosphere. Appl. Geochem..

[57] Kietäväinen R., Ahonen L., Kukkonen I.T., Niedermann S., Wiersberg T. (2014). Noble gas residence times of saline waters within crystalline bedrock, Outokumpu Deep Drill Hole, Finland. Geochim. Cosmochim. Acta.

[58] Nuppunen-Puputti M., Purkamo L., Kietäväinen R., Nyyssönen M., Itävaara M., Ahonen L., Kukkonen I., Bomberg M. (2018). Rare Biosphere Archaea Assimilate Acetate in Precambrian Terrestrial Subsurface at 2.2 km Depth. Geosciences.

[59] Purkamo L., Bomberg M., Kietäväinen R., Salavirta H., Nyyssönen M., Nuppunen-Puputti M., Ahonen L., Kukkonen I., Itävaara M. (2016). Microbial co-occurrence patterns in deep Precambrian bedrock fracture fluids. Biogeosciences.

[60] Lahtinen R., Huhma H., Kontinen A., Kohonen J., Sorjonen-Ward P. (2010). New constraints for the source characteristics, deposition and age of the 2.1–1.9 Ga metasedimentary cover at the western margin of the Karelian Province. Precambrian Res..

[61] Purkamo L., Bomberg M., Nyyssönen M., Ahonen L., Kukkonen I., Itävaara M. (2017). Response of Deep Subsurface Microbial Community to Different Carbon Sources and Electron Acceptors during ∼2 months Incubation in Microcosms. Front. Microbiol..

[62] Herlemann D.P.R., Labrenz M., Jürgens K., Bertilsson S., Waniek J.J., Andersson A.F. (2011). Transitions in bacterial communities along the 2000 km salinity gradient of the Baltic Sea. ISME J..

[63] Klindworth A., Pruesse E., Schweer T., Peplies J., Quast C., Horn M., Glöckner F.O. (2013). Evaluation of general 16S ribosomal RNA gene PCR primers for classical and next-generation sequencing-based diversity studies. Nucleic Acids Res..

[64] Gardes M., Bruns T.D. (1993). ITS primers with enhanced specificity for basidiomycetes—Application to the identification of mycorrhizae and rusts. Mol. Ecol..

[65] Schloss P.D., Westcott S.L., Ryabin T., Hall J.R., Hartmann M., Hollister E.B., Lesniewski R.A., Oakley B.B., Parks D.H., Robinson C.J. (2009). Introducing mothur: Open-Source, Platform-Independent, Community-Supported Software for Describing and Comparing Microbial Communities. Appl. Environ. Microbiol..

[66] Quast C., Pruesse E., Yilmaz P., Gerken J., Schweer T., Yarza P., Peplies J., Glöckner F.O. (2013). The SILVA ribosomal RNA gene database project: Improved data processing and web-based tools. Nucleic Acids Res..

[67] Glöckner F.O., Yilmaz P., Quast C., Gerken J., Beccati A., Ciuprina A., Bruns G., Yarza P., Peplies J., Westram R. (2017). 25 years of serving the community with ribosomal RNA gene reference databases and tools. J. Biotechnol..

[68] Kõljalg U., Nilsson R.H., Abarenkov K., Tedersoo L., Taylor A.F.S., Bahram M., Bates S.T., Bruns T.D., Bengtsson-Palme J., Callaghan T.M. (2013). Towards a unified paradigm for sequence-based identification of fungi. Mol. Ecol..

[69] Nilsson R.H., Larsson K.-H., Taylor A.F.S., Bengtsson-Palme J., Jeppesen T.S., Schigel D., Kennedy P., Picard K., Glöckner F.O., Tedersoo L. (2019). The UNITE database for molecular identification of fungi: Handling dark taxa and parallel taxonomic classifications. Nucleic Acids Res..

[70] Caporaso J.G., Kuczynski J., Stombaugh J., Bittinger K., Bushman F.D., Costello E.K., Fierer N., Peña A.G., Goodrich J.K., Gordon J.I. (2010). QIIME allows analysis of high-throughput community sequencing data. Nat. Methods.

[71] Comeau A.M., Douglas G.M., Langille M.G.I. (2017). Microbiome Helper: A Custom and Streamlined Workflow for Microbiome Research. mSystems.

[72] RStudio Team (2015). RStudio: Integrated Development for R.

[73] McMurdie P.J., Holmes S. (2013). phyloseq: An R Package for Reproducible Interactive Analysis and Graphics of Microbiome Census Data. PLoS ONE.

[74] Oksanen J., Blanchet F.G., Friendly M., Kindt R., Legendre P., McGlinn D., Minchin P.R., O’Hara R.B., Simpson G.L., Solymos P. Vegan: Community Ecology Package. 2018. https://CRAN.R-project.org/package=vegan.

[75] Wickham H. (2016). Ggplot2: Elegant Graphics for Data Analysis.

[76] Ntarlagiannis D., Atekwana E.A., Hill E.A., Gorby Y. (2007). Microbial nanowires: Is the subsurface “hardwired”?. Geophys. Res. Lett..

[77] Pospíšil J., Vítovská D., Kofroňová O., Muchová K., Šanderová H., Hubálek M., Šiková M., Modrak M., Benada O., Barak I. (2020). Bacterial nanotubes as a manifestation of cell death. Nat. Commun..

[78] Marguet E., Gaudin M., Gauliard E., Fourquaux I., Plouy S.L.B.D., Matsui I., Forterre P. (2013). Membrane vesicles, nanopods and/or nanotubes produced by hyperthermophilic archaea of the genus Thermococcus. Biochem. Soc. Trans..

[79] Loukola-Ruskeeniemi K., Kukkonen I.T. (2011). Graphite- and sulphide-bearing schists in the Outokumpu R2500 Drill Core: Comparison with the Ni-Cu-Co-Zn-Mn-rich black schists at Talvivaara, Finland. Geological Survey of Finland, Special Paper 51. Outokumpu Deep Drilling Project 2003–2010.

[80] Taran L.N., Onoshko M.P., Mikhailov N.D., Kukkonen I.T. (2011). Structure and composition of organic matter and isotope geochemistry of the palaeoproterozoic graphite and sulphide-rich metasedimentary rocks from the Outokumpu Deep Drill Hole, eastern Finland. Geological Survey of Finland, Special Paper 51. Outokumpu Deep Drilling Project 2003–2010.

[81] D’Hondt S., Rutherford S., Spivack A.J. (2002). Metabolic Activity of Subsurface Life in Deep-Sea Sediments. Science.

[82] Fredrickson J.K., Balkwill D.L. (2006). Geomicrobial Processes and Biodiversity in the Deep Terrestrial Subsurface. Geomicrobiol. J..

[83] Lau M.C.Y., Kieft T.L., Kuloyo O., Linage-Alvarez B., Van Heerden E., Lindsay M.R., Magnabosco C., Wang W., Wiggins J.B., Guo L. (2016). An oligotrophic deep-subsurface community dependent on syntrophy is dominated by sulfur-driven autotrophic denitrifiers. Proc. Natl. Acad. Sci. USA.

[84] Flemming H.-C., Stefan W. (2019). Bacteria and archaea on Earth and their abundance in biofilms. Nat. Rev. Microbiol..

[85] MacLean L.C.W., Pray T.J., Onstott T.C., Brodie E.L., Hazen T.C., Southam G. (2007). Mineralogical, Chemical and Biological Characterization of an Anaerobic Biofilm Collected from a Borehole in a Deep Gold Mine in South Africa. Geomicrobiol. J..

[86] Saitoh Y., Hirano S.-I., Nagaoka T., Amano Y. (2019). Genetic survey of indigenous microbial eukaryotic communities, mainly fungi, in sedimentary rock matrices of deep terrestrial subsurface. Ecol. Genet. Genom..

[87] Abena M.T.B., Chen G., Chen Z., Zheng X., Li S., Li T., Zhong W. (2020). Microbial diversity changes and enrichment of potential petroleum hydrocarbon degraders in crude oil-, diesel-, and gasoline-contaminated soil. 3 Biotech.

[88] Held B.W., Salomon C.E., Blanchette R.A. (2020). Diverse subterranean fungi of an underground iron ore mine. PLoS ONE.

[89] Sato Y., Narisawa K., Tsuruta K., Umezu M., Nishizawa T., Tanaka K., Yamaguchi K., Komatsuzaki M., Ohta H. (2010). Detection of Betaproteobacteria inside the Mycelium of the Fungus Mortierella elongata. Microbes Environ..

[90] Liu X.-Z., Wang Q.-M., Goker M., Groenewald M., Kachalkin A.V., Lumbsch H.T., Millanes A.M., Wedin M., Yurkov A.M., Boekhout T. (2015). Towards an integrated phylogenetic classification of the Tremellomycetes. Stud. Mycol..

[91] Tsuji M., Tanabe Y., Vincent W.F., Uchida M. (2019). Vishniacozyma ellesmerensis sp. nov., a psychrophilic yeast isolated from a retreating glacier in the Canadian High Arctic. Int. J. Syst. Evol. Microbiol..

[92] Ogaki M.B., Teixeira D.R., Vieira R., Lirio J.M., Felizardo J.P., Abuchacra R.C., Cardoso R.P., Zani C.L., Alves T.M.A., Junior P.A.S. (2020). Diversity and bioprospecting of cultivable fungal assemblages in sediments of lakes in the Antarctic Peninsula. Fungal Biol..

[93] Schmidt S.K., Vimercati L., Darcy J.L., Arán P., Gendron E.M.S., Solon A.J., Porazinska D., Dorador C. (2017). A *Naganishia* in high places: Functioning populations or dormant cells from the atmosphere?. Mycology.

[94] Musa H., Kasim F.H., Gunny A.A.N., Gopinath S.C.B. (2018). Salt-adapted moulds and yeasts: Potentials in industrial and environmental biotechnology. Process Biochem..

[95] Takii S., Hanada S., Tamaki H., Ueno Y., Sekiguchi Y., Ibe A., Matsuura K. (2007). Dethiosulfatibacter aminovorans gen. nov., sp. nov., a novel thiosulfate-reducing bacterium isolated from coastal marine sediment via sulfate-reducing enrichment with Casamino acids. Int. J. Syst. Evol. Microbiol..

[96] Sorokin D.Y., Tourova T.P., Mußmann M., Muyzer G. (2008). Dethiobacter alkaliphilus gen. nov. sp. nov., and Desulfurivibrio alkaliphilus gen. nov. sp. nov.: Two novel representatives of reductive sulfur cycle from soda lakes. Extremophiles.

[97] Hoefs J. (2015). Isotope Fractionation Processes of Selected Elements. Stable Isotope Geochemistry.

[98] Drake H., Ivarsson M. (2018). The role of anaerobic fungi in fundamental biogeochemical cycles in the deep biosphere. Fungal Biol. Rev..

[99] Ivarsson M., Schnürer A., Bengtson S., Neubeck A. (2016). Anaerobic Fungi: A Potential Source of Biological H2 in the Oceanic Crust. Front. Microbiol..

[100] Spinnler H.-E., Berger C., Lapadatescu C., Bonnarme P. (2001). Production of sulfur compounds by several yeasts of technological interest for cheese ripening. Int. Dairy J..

[101] Buzzini P., Romano S., Turchetti B., Vaughan A., Pagnoni U.M., Davoli P. (2005). Production of volatile organic sulfur compounds (VOSCs) by basidiomycetous yeasts. FEMS Yeast Res..

[102] Clark I., Fritz P. (1997). Environmental Isotopes in Hydrogeology.

[103] Kloppmann W., Girard J.-P., Négrel P. (2002). Exotic stable isotope compositions of saline waters and brines from the crystalline basement. Chem. Geol..

[104] Rose T., Télouk P., Fiebig J., Marschall H.R., Klein S. (2020). Iron and oxygen isotope systematics during corrosion of iron objects: A first approach. Archaeol. Anthr. Sci..

[105] Lin L.-H., Slater G.F., Lollar B.S., Lacrampe-Couloume G., Onstott T. (2005). The yield and isotopic composition of radiolytic H2, a potential energy source for the deep subsurface biosphere. Geochim. Cosmochim. Acta.

