# Supplementary material for: Rock Surface Fungi in Deep Continental Biosphere—Exploration of Microbial Community Formation with Subsurface In Situ Biofilm Trap
Source: Microorganisms. 2020 Dec 29;9(1):64. doi: 10.3390/microorganisms9010064 (PMC7824546; doi:10.3390/microorganisms9010064)
Supplement: Supplementary file 1 [file microorganisms-09-00064-s001.zip › Supplementary_tables_and_figures.docx]

Supplementary file for “Rock Surface Fungi in Deep Continental Biosphere—Exploration of Microbial Community Formation with Subsurface In Situ Biofilm Trap”

**Maija Nuppunen-Puputti, Riikka Kietäväinen, Lotta Purkamo, Pauliina Rajala, Merja Itävaara, Ilmo Kukkonen and Malin Bomberg**

This supplementary file contains additional figures and tables supporting the manuscript.

**Figure S2 (a-e).** Inner parts of the *in situ* biofilm trap

#####
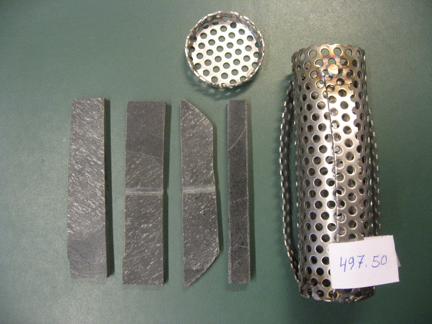


##### a) Sample cage and mica schist slides for scanning electron microscopy


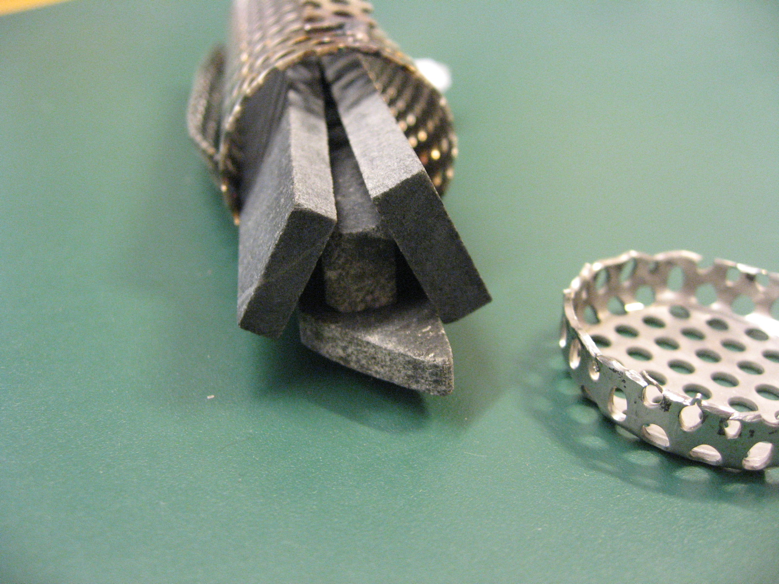


##### b) Sample cage and inserted mica schist slides for scanning electron microscopy. Center piece of mica schist

##### keeps the slides apart enabling biofilm formation on the surfaces.


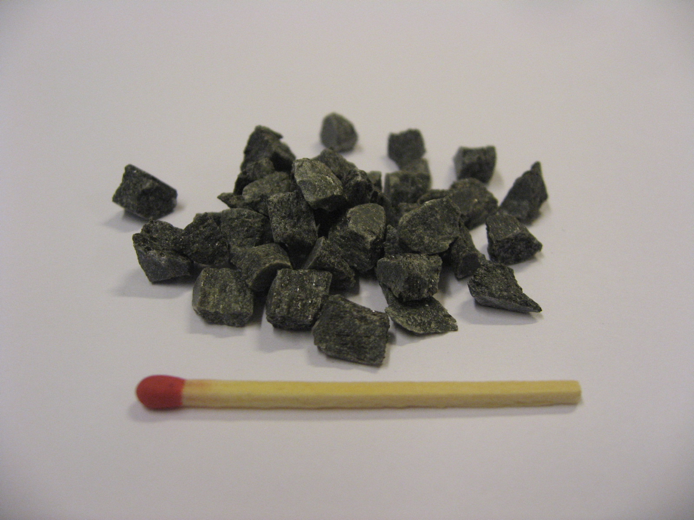


##### c) Crushed mica schist prior to heat treatment


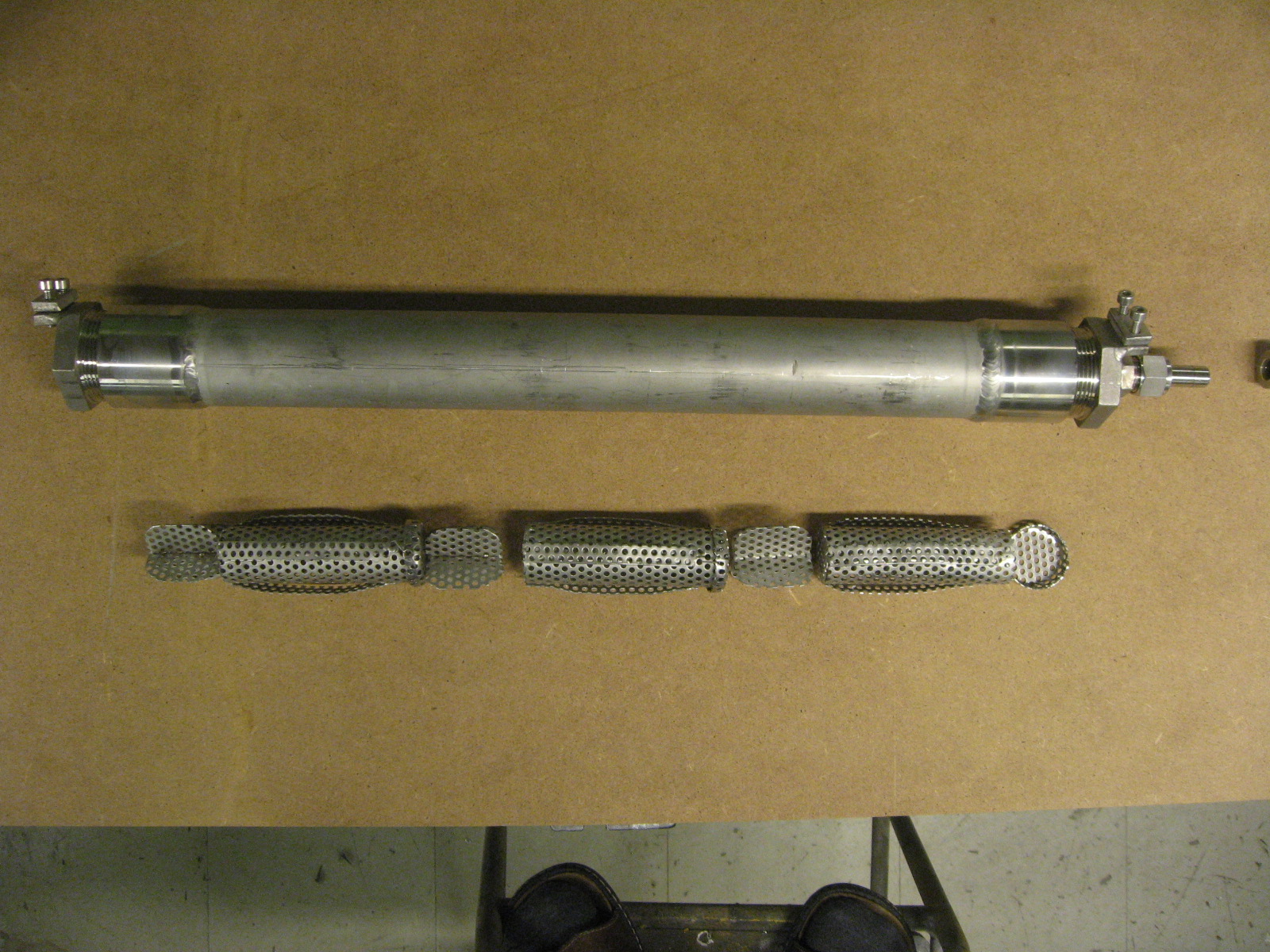


d) *In situ* biofilm trap cabinet (at the top) and inner sample cages (at the bottom) containing sample surface materials. In addition, separators between the sample cages.


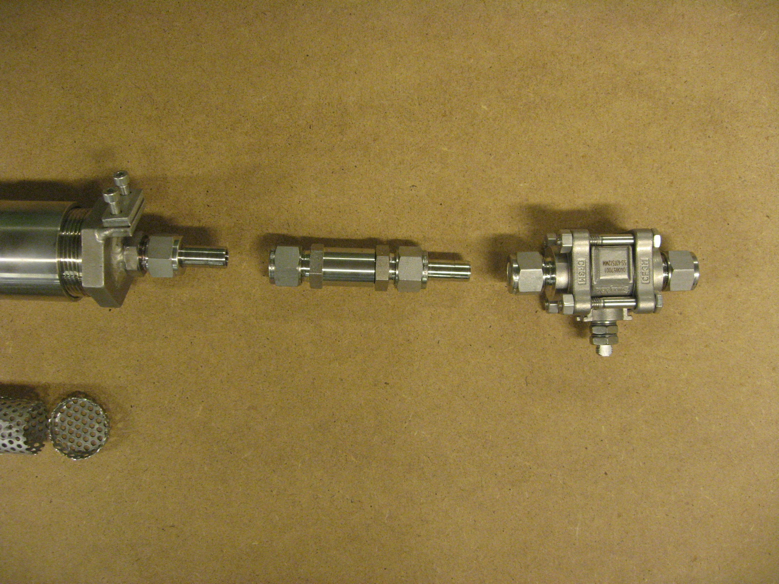


e) Further visualization on connecting parts of the *in situ* biofilm trap


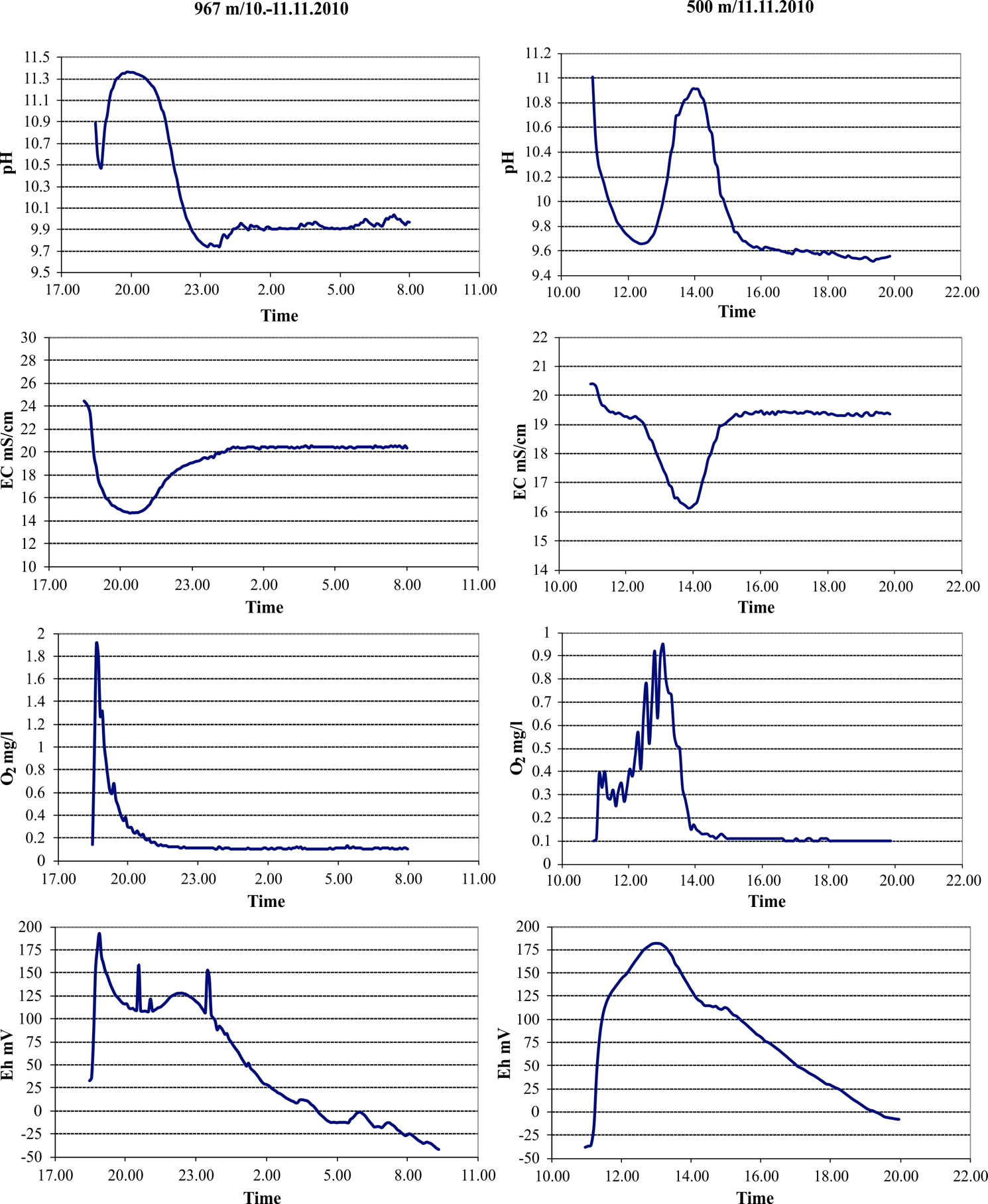


**FigureS3.** Online monitoring parametres for pH, electrical conductivity (EC), dissolved O_2_ and redox potential (Eh vs. SHE) for water pumped through the traps from the depth of 500 m (right) or the depth of 967 m (left) before initiation of the incubation period.


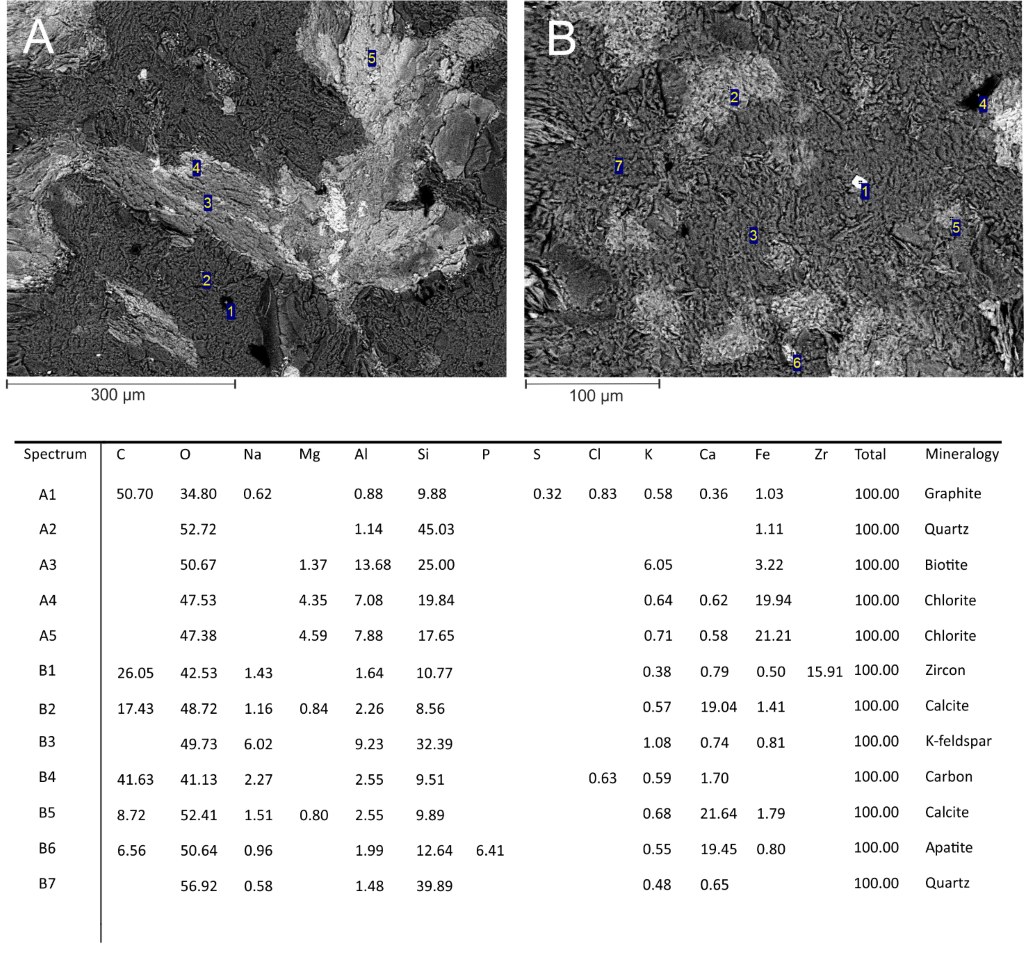


**Figure S4.** Examples of back scattered electron (BSE) images, chemical composition in weight-%, and interpreted mineralogy of mica schist plates from 500 m depth before the biofilm trap experiment. Data is normalized to 100 %. Note that uncoated samples and spot size of 50 µm does not allow quantitative and detailed analysis of the samples.


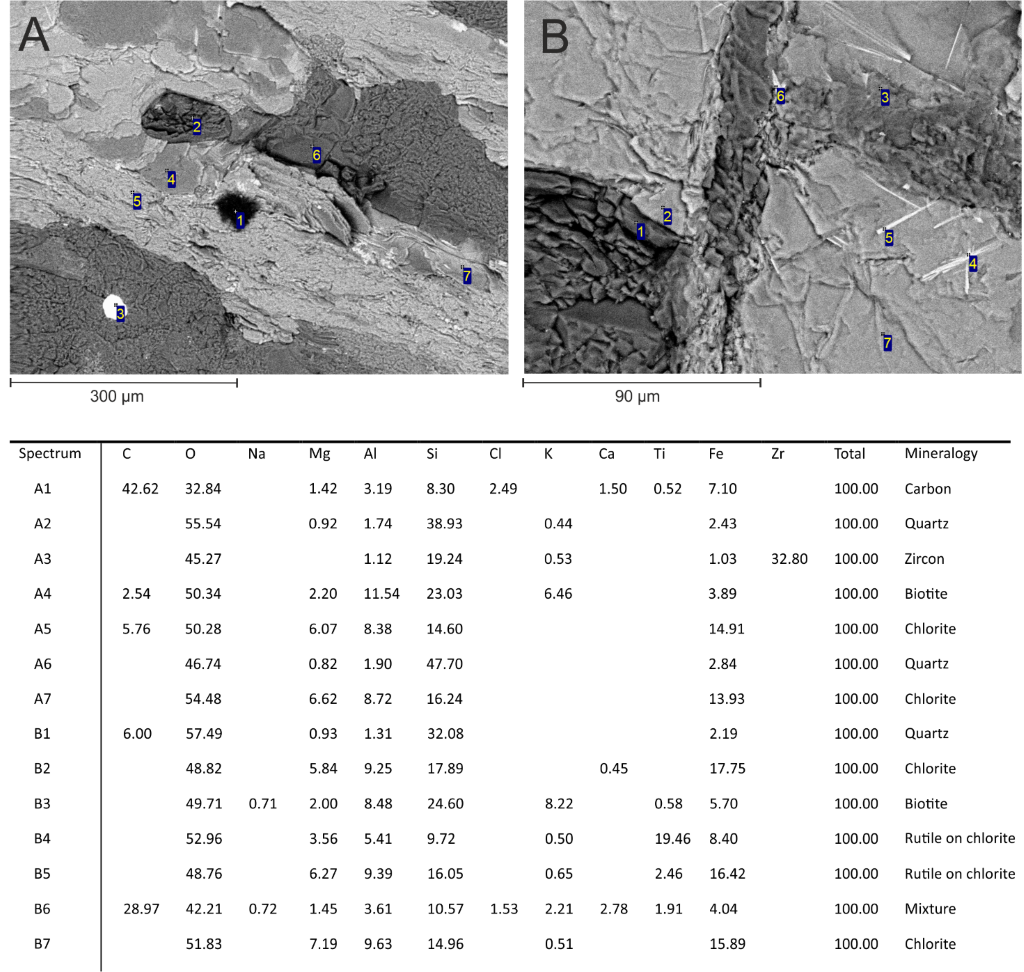


**Figure S5.** Examples of back scattered electron (BSE) images, chemical composition in weight-%, and interpreted mineralogy of mica schist plates from 500 m depth after the biofilm trap experiment. Data is normalized to 100 %. Note that uncoated samples and spot size of 50 µm does not allow quantitative and detailed analysis of the samples.

#### **Table S2 (a-b).** Alphadiversity measures of fungal (a) and bacterial (b) communities from raw data displaying number of sequences, observed species, richness estimators (Chao1, ACE) and diversity indices (Shannon, Simpson). Chao1 and ACE estimates include a standard error, se.chao1 and se.ACE.

a)

| Alphadiversity measures for the fungal communities | | | | | | | |
| --- | --- | --- | --- | --- | --- | --- | --- |
|  | **Observed** | **Chao1** | **se.chao1** | **ACE** | **se.ACE** | **Shannon** | **Simpson** |
| trap_500m_closed_glass | 170 | 340.6 | 51.3 | 320.2 | 10.7 | 2.8 | 0.8 |
| trap_500m_closed_rock_A | 69 | 161.6 | 44.0 | 170.7 | 7.9 | 2.3 | 0.8 |
| trap_500m_closed_rock_B | 142 | 506.2 | 127.3 | 348.6 | 10.1 | 2.8 | 0.9 |
| trap_500m_closed_rock_C | 147 | 317.1 | 54.4 | 304.1 | 10.0 | 2.5 | 0.8 |
| trap_500m_closed_water_A | 87 | 189.7 | 39.0 | 186.7 | 5.9 | 1.8 | 0.7 |
| trap_500m_closed_water_B | 248 | 386.2 | 37.1 | 392.0 | 11.2 | 2.9 | 0.8 |
| trap_500m_open_glass | 275 | 516.0 | 54.8 | 566.8 | 16.0 | 3.3 | 0.9 |
| trap_500m_open_rock_A | 124 | 232.5 | 36.7 | 281.8 | 11.6 | 2.9 | 0.9 |
| trap_500m_open_rock_B | 193 | 505.1 | 90.3 | 458.9 | 14.6 | 3.6 | 0.9 |
| trap_500m_open_rock_C | 187 | 341.7 | 43.0 | 383.1 | 12.2 | 2.1 | 0.6 |
| trap_967m_closed_glass | 94 | 204.6 | 40.7 | 236.2 | 10.3 | 2.5 | 0.8 |
| trap_967m_closed_rock_A | 184 | 369.5 | 51.0 | 403.5 | 11.5 | 3.3 | 0.9 |
| trap_967m_closed_rock_B | 123 | 598.3 | 180.6 | 511.3 | 13.2 | 2.3 | 0.8 |
| trap_967m_closed_rock_C | 81 | 148.6 | 28.2 | 173.8 | 9.5 | 2.4 | 0.7 |
| trap_967m_closed_water_A | 215 | 520.0 | 83.1 | 511.3 | 14.7 | 3.4 | 0.9 |
| trap_967m_closed_water_B | 191 | 326.6 | 36.5 | 351.4 | 11.9 | 3.2 | 0.9 |
| trap_967m_open_glass | 229 | 516.0 | 76.7 | 455.2 | 12.7 | 3.3 | 0.9 |
| trap_967m_open_rock_A | 174 | 347.9 | 47.3 | 417.6 | 13.2 | 1.6 | 0.6 |
| trap_967m_open_rock_B | 112 | 307.5 | 73.2 | 270.9 | 10.5 | 3.3 | 0.9 |
| trap_967m_open_rock_C | 176 | 311.3 | 38.1 | 384.6 | 13.8 | 3.7 | 0.9 |

b)

| Alphadiversity measures for the bacterial communities | | | | | | | |
| --- | --- | --- | --- | --- | --- | --- | --- |
|  | **Observed** | **Chao1** | **se.chao1** | **ACE** | **se.ACE** | **Shannon** | **Simpson** |
| trap_500m_closed_glass | 96 | 189.9 | 34.8 | 221.3 | 8.0 | 2.5 | 0.8 |
| trap_500m_closed_rock_A | 52 | 89.8 | 19.4 | 107.0 | 5.4 | 1.8 | 0.7 |
| trap_500m_closed_rock_B | 32 | 62.0 | 20.9 | 80.4 | 5.4 | 2.4 | 0.9 |
| trap_500m_closed_rock_C | 67 | 164.5 | 45.9 | 219.5 | 9.7 | 2.4 | 0.9 |
| trap_500m_closed_water_A | 48 | 85.5 | 20.6 | 111.0 | 6.7 | 2.5 | 0.8 |
| trap_500m_closed_water_B | 105 | 235.1 | 45.7 | 314.2 | 11.2 | 2.6 | 0.9 |
| trap_500m_open_glass | 65 | 151.1 | 38.4 | 209.6 | 9.7 | 1.7 | 0.7 |
| trap_500m_open_rock_A | 135 | 305.5 | 55.4 | 357.5 | 12.0 | 3.0 | 0.9 |
| trap_500m_open_rock_B | 51 | 126.6 | 42.8 | 108.5 | 6.1 | 1.9 | 0.7 |
| trap_500m_open_rock_C | 47 | 61.2 | 8.7 | 72.3 | 5.0 | 2.1 | 0.7 |
| trap_967m_closed_glass | 70 | 144.0 | 35.0 | 150.7 | 8.2 | 2.8 | 0.9 |
| trap_967m_closed_rock_A | 66 | 222.0 | 80.9 | 186.7 | 9.0 | 2.4 | 0.9 |
| trap_967m_closed_rock_B | 88 | 154.4 | 26.2 | 166.2 | 5.6 | 2.6 | 0.9 |
| trap_967m_closed_rock_C | 80 | 241.1 | 74.5 | 217.3 | 8.9 | 2.3 | 0.8 |
| trap_967m_closed_water_A | 81 | 208.5 | 53.8 | 275.8 | 11.7 | 2.5 | 0.9 |
| trap_967m_closed_water_B | 135 | 432.5 | 106.2 | 348.0 | 9.1 | 3.0 | 0.9 |
| trap_967m_open_glass | 72 | 135.9 | 29.0 | 161.8 | 9.4 | 2.1 | 0.8 |
| trap_967m_open_rock_A | 69 | 198.0 | 61.2 | 267.0 | 10.9 | 2.3 | 0.8 |
| trap_967m_open_rock_B | 68 | 173.9 | 51.5 | 161.3 | 7.6 | 2.3 | 0.8 |
| trap_967m_open_rock_C | 75 | 169.6 | 41.6 | 190.9 | 8.8 | 2.3 | 0.8 |

**Table S3 a-b.** Library sizes for fungal (a) and bacterial (b) communities

1. Fungal

|  | Library size |
| --- | --- |
| trap_500m_closed_glass | 1630 |
| trap_500m_closed_rock_A | 1258 |
| trap_500m_closed_rock_B | 1063 |
| trap_500m_closed_rock_C | 2131 |
| trap_500m_closed_water_A | 1593 |
| trap_500m_closed_water_B | 4020 |
| trap_500m_open_glass | 2542 |
| trap_500m_open_rock_A | 1808 |
| trap_500m_open_rock_B | 1344 |
| trap_500m_open_rock_C | 2425 |
| trap_967m_closed_glass | 901 |
| trap_967m_closed_rock_A | 2065 |
| trap_967m_closed_rock_B | 1554 |
| trap_967m_closed_rock_C | 698 |
| trap_967m_closed_water_A | 3257 |
| trap_967m_closed_water_B | 1979 |
| trap_967m_open_glass | 2793 |
| trap_967m_open_rock_A | 4019 |
| trap_967m_open_rock_B | 689 |
| trap_967m_open_rock_C | 1216 |

1. Bacterial

|  | Library size |
| --- | --- |
| trap_500m_closed_glass | 1280 |
| trap_500m_closed_rock_A | 1053 |
| trap_500m_closed_rock_B | 453 |
| trap_500m_closed_rock_C | 1491 |
| trap_500m_closed_water_A | 489 |
| trap_500m_closed_water_B | 2348 |
| trap_500m_open_glass | 1508 |
| trap_500m_open_rock_A | 2309 |
| trap_500m_open_rock_B | 939 |
| trap_500m_open_rock_C | 635 |
| trap_967m_closed_glass | 936 |
| trap_967m_closed_rock_A | 1086 |
| trap_967m_closed_rock_B | 1472 |
| trap_967m_closed_rock_C | 1429 |
| trap_967m_closed_water_A | 1806 |
| trap_967m_closed_water_B | 2182 |
| trap_967m_open_glass | 1463 |
| trap_967m_open_rock_A | 1977 |
| trap_967m_open_rock_B | 1359 |
| trap_967m_open_rock_C | 1534 |

#### **Figure S6 (a-g).** Additional taxonomy figures for Bacteria. In detail: a) Actinobacteria, b) Bacteroidota, c) Desulfobacterota, d) Dethiobacter, e) Cyanobacteria, f) Firmicutes, g) Gammaproteobacteria.


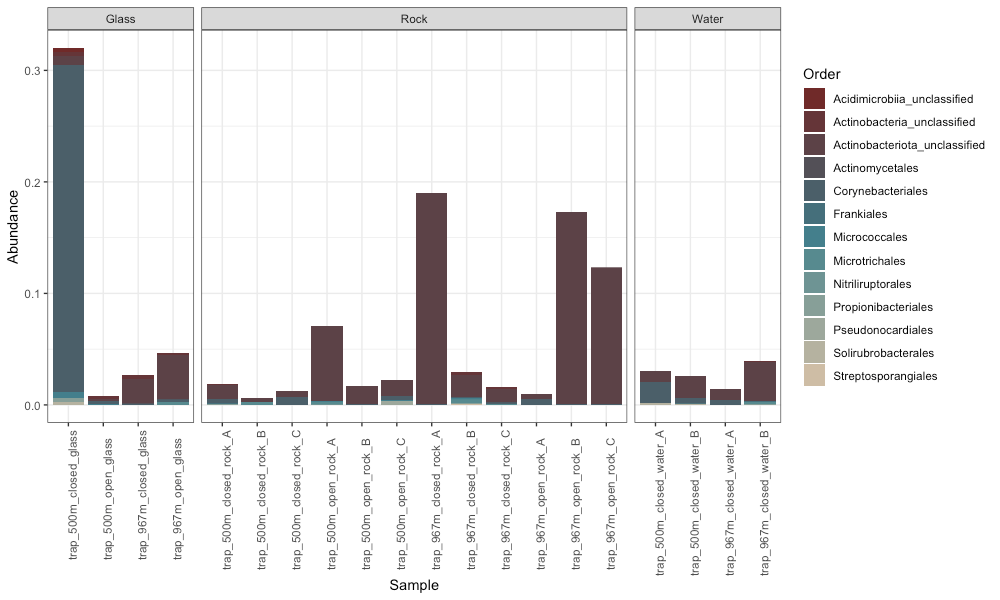


1. Actinobacter (Order level)


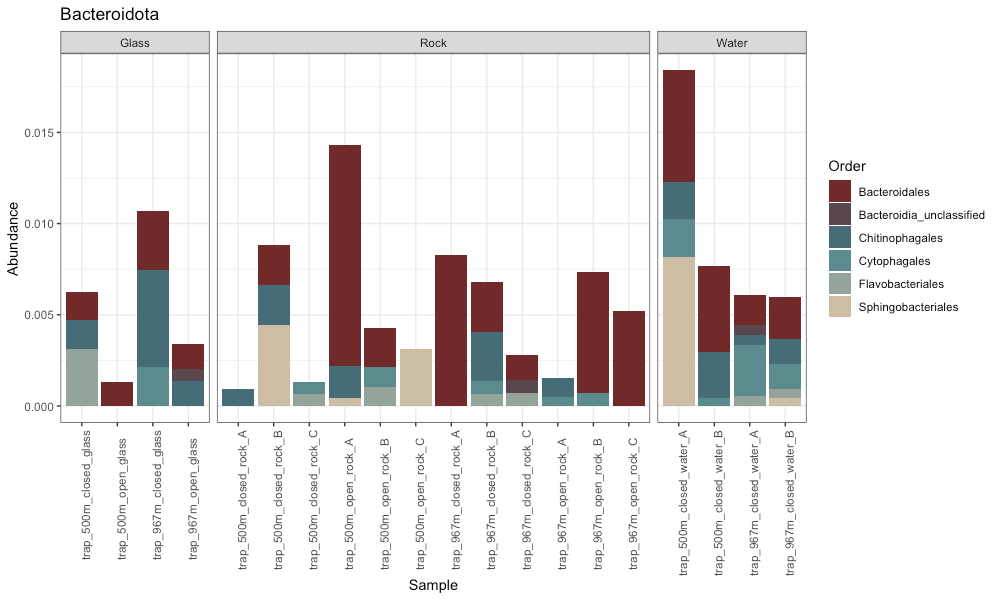


1. Bacteroidota (Order level)


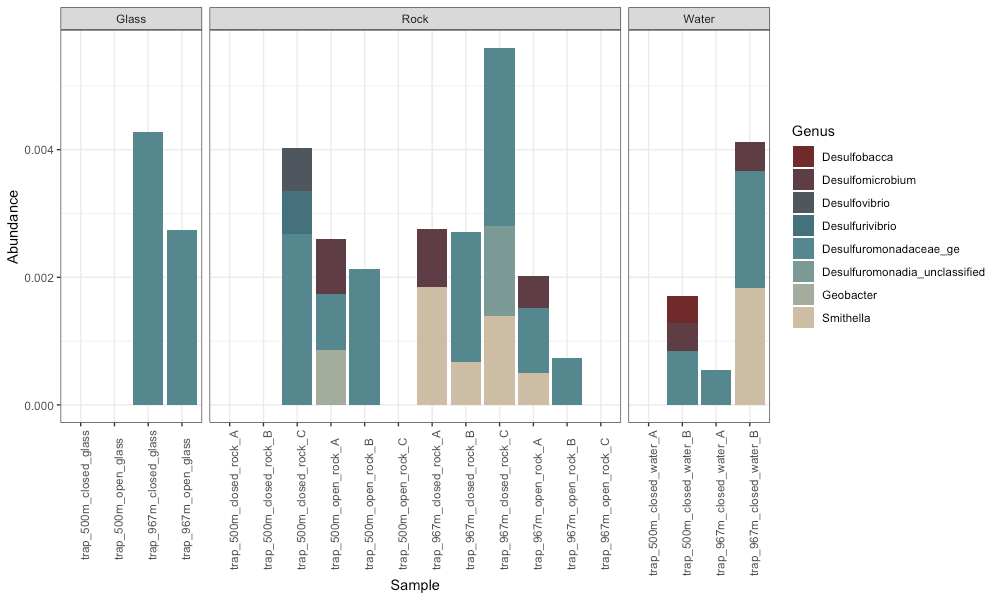


1. Desulfobacterota (Genus level)


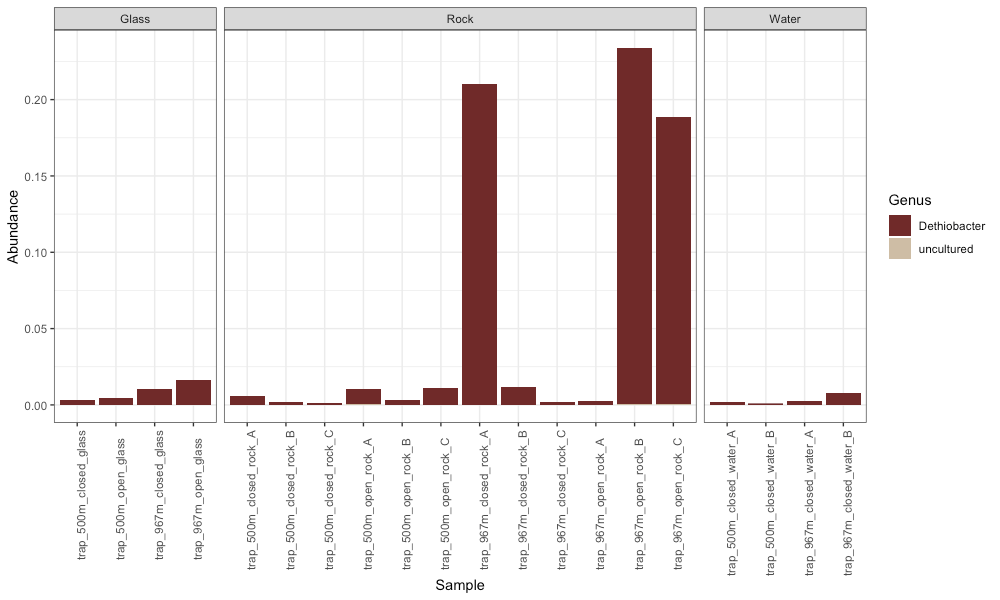


1. Dethiobacteria (Genus level)


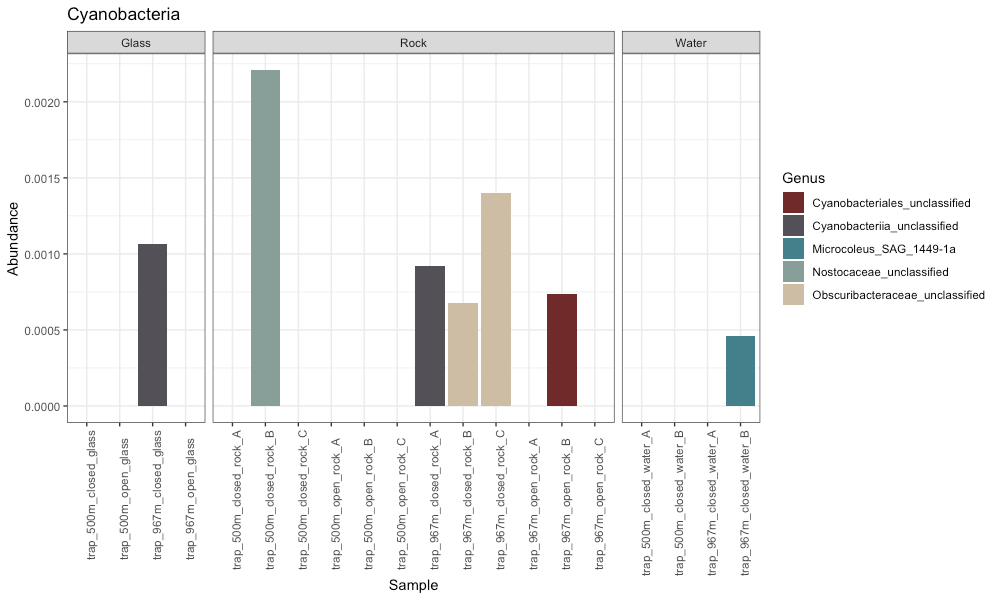


1. Cyanobacteria (Genus level)


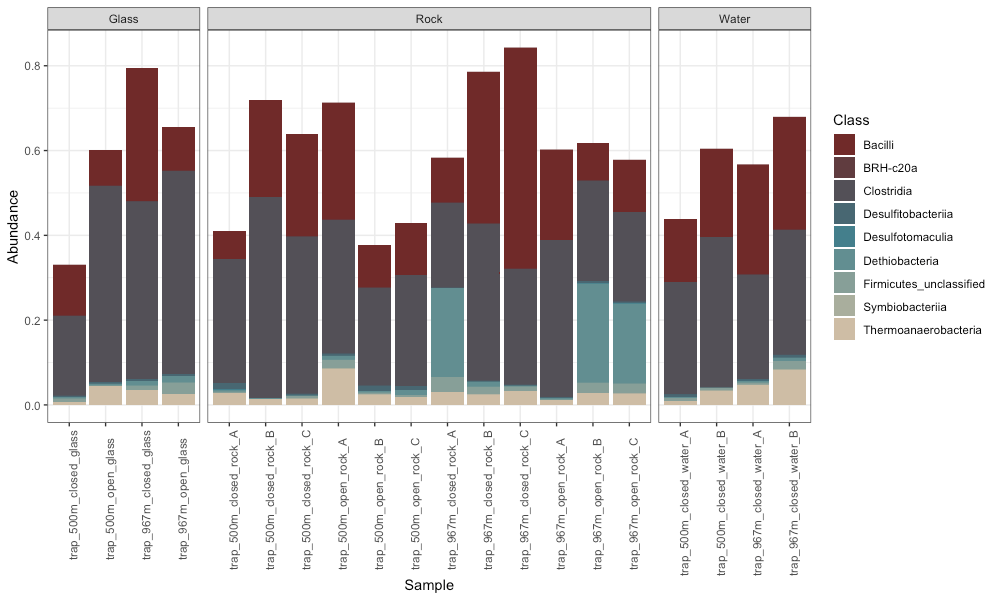


1. Firmicutes (Class level)


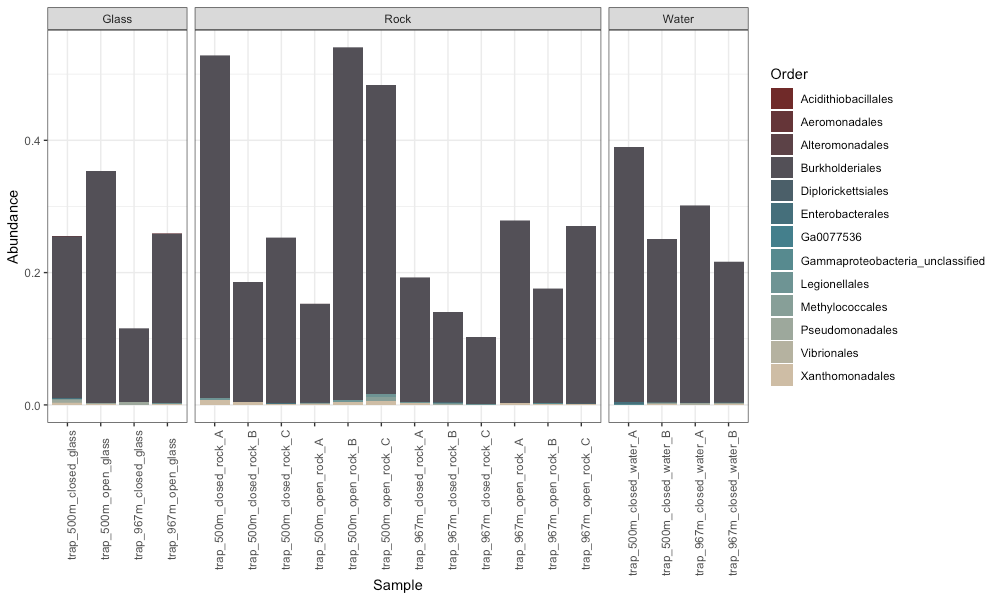


1. Gammaproteobacteria (Order level)

**Table S6 (a-c).** SPARCC-correlations between rock surface microbial communities for a) Bacteria - Fungi, b) Fungi - Fungi, c) Bacteria - Bacteria.

1. **Bacteria-Fungi**

| Bacteria | Fungi | Correlation | p-value |
| --- | --- | --- | --- |
| Acholeplasmataceae_unclassified_Otu0022 | p__Ascomycota_unclassified_Otu0075 | -0.687224 | 0 |
| Acholeplasmataceae_unclassified_Otu0022 | f__Herpotrichiellaceae_unclassified_Otu0098 | -0.619528 | 0 |
| Acholeplasmataceae_unclassified_Otu0022 | p__Ascomycota_unclassified_Otu0076 | -0.5903 | 0 |
| Acholeplasmataceae_unclassified_Otu0022 | g__Knufia_unclassified_Otu0055 | -0.573489 | 0 |
| Acholeplasmataceae_unclassified_Otu0022 | s__Vishniacozyma_victoriae_Otu0064 | -0.524992 | 0.012 |
| Acholeplasmataceae_unclassified_Otu0022 | f__Aureobasidiaceae_unclassified_Otu0035 | 0.517806 | 0.024 |
| Comamonadaceae_unclassified_Otu0016 | s__Tremellomycetes_sp_Otu0013 | 0.57823 | 0.012 |
| Comamonadaceae_unclassified_Otu0016 | s__Naganishia_friedmannii_Otu0030 | 0.545466 | 0.024 |
| Comamonadaceae_unclassified_Otu0016 | g__Perusta_unclassifiedOtu0083 | 0.551872 | 0.036 |
| Dethiobacter_Otu0012 | p__Ascomycota_unclassified_Otu0075 | 0.532361 | 0.025 |
| Dethiosulfatibacter_Otu0017 | p__Ascomycota_unclassified_Otu0075 | -0.52858 | 0 |
| Dethiosulfatibacter_Otu0017 | g__Ramularia_unclassified_Otu0026 | 0.664087 | 0.024 |
| Erysipelothrix_Otu0033 | s__Debaryomyces_hansenii_Otu0019 | 0.611261 | 0.036 |
| Erysipelothrix_Otu0056 | f__Nectriaceae_unclassified_Otu0033 | -0.548144 | 0 |
| Erysipelothrix_Otu0056 | s__Dioszegia_hungarica_Otu0088 | 0.537447 | 0.024 |
| Erysipelothrix_Otu0060 | c__Dothideomycetes_unclassified_Otu0067 | -0.640331 | 0 |
| Erysipelothrix_Otu0060 | s__Naganishia_friedmannii_Otu0030 | -0.591695 | 0 |
| Erysipelothrix_Otu0060 | s__Sarocladium_bactrocephalum_Otu0056 | -0.554321 | 0.024 |
| Erysipelothrix_Otu0060 | s__Debaryomyces_hansenii_Otu0019 | 0.55117 | 0.037 |
| Lachnospirales_unclassified_Otu0058 | s__Debaryomyces_hansenii_Otu0019 | 0.625826 | 0.025 |
| Proteiniclasticum_Otu0059 | s__Vishniacozyma_victoriae_Otu0064 | -0.60042 | 0 |
| Proteiniclasticum_Otu0059 | s__Debaryomyces_hansenii_Otu0019 | 0.596472 | 0 |
| Proteiniclasticum_Otu0059 | f__Herpotrichiellaceae_unclassified_Otu0098 | -0.520487 | 0 |
| Proteiniclasticum_Otu0059 | g__Knufia_unclassified_Otu0055 | -0.518795 | 0 |
| Proteiniclasticum_Otu0059 | p__Ascomycota_unclassified_Otu0075 | -0.56876 | 0.012 |
| Proteiniclasticum_Otu0059 | p__Ascomycota_unclassified_Otu0076 | -0.534273 | 0.012 |
| Rhodobacteraceae_unclassified_Otu0018 | f__Herpotrichiellaceae_unclassified_Otu0098 | -0.684138 | 0 |
| Rhodobacteraceae_unclassified_Otu0018 | g__Knufia_unclassified_Otu0055 | -0.619922 | 0 |
| Rhodobacteraceae_unclassified_Otu0018 | s__Vishniacozyma_victoriae_Otu0064 | -0.544463 | 0 |
| Rhodobacteraceae_unclassified_Otu0018 | p__Ascomycota_unclassified_Otu0075 | -0.655796 | 0.012 |
| Rhodobacteraceae_unclassified_Otu0018 | p__Ascomycota_unclassified_Otu0076 | -0.620232 | 0.012 |
| Rhodobacteraceae_unclassified_Otu0035 | f__Didymellaceae_unclassified_Otu0011 | 0.849439 | 0 |
| Rhodobacteraceae_unclassified_Otu0035 | g__Ramularia_unclassified_Otu0026 | 0.707549 | 0 |
| Rhodobacteraceae_unclassified_Otu0035 | g__Mortierella_unclassified_Otu0004 | 0.514285 | 0.024 |
| Rhodobacteraceae_unclassified_Otu0035 | f__Didymellaceae_unclassified_Otu0063 | 0.500085 | 0.025 |
| Soehngenia_Otu0057 | g__Perusta_unclassifiedOtu0083 | -0.598909 | 0 |
| Soehngenia_Otu0057 | s__Debaryomyces_hansenii_Otu0019 | 0.563689 | 0.036 |
| SRB2_ge_Otu0023 | f__Didymellaceae_unclassified_Otu0063 | -0.582967 | 0 |
| SRB2_ge_Otu0023 | f__Nectriaceae_unclassified_Otu0033 | -0.59481 | 0.012 |
| SRB2_ge_Otu0023 | g__Phacidium_unclassified_Otu0042 | 0.543846 | 0.048 |
| SRB2_ge_Otu0063 | s__Debaryomyces_hansenii_Otu0019 | 0.719945 | 0 |
| SRB2_ge_Otu0063 | f__Aplosporellaceae_unclassified_Otu0047 | 0.689496 | 0.012 |
| SRB2_ge_Otu0063 | g__Sarocladium_unclassified_Otu0028 | 0.620466 | 0.013 |
| SRB2_ge_Otu0063 | s__Phoma_multirostrata_Otu0029 | 0.544537 | 0.048 |
| SRB2_ge_Otu0064 | s__Debaryomyces_hansenii_Otu0019 | 0.680391 | 0 |
| SRB2_ge_Otu0064 | f__Didymellaceae_unclassified_Otu0011 | -0.549408 | 0 |
| SRB2_ge_Otu0064 | f__Aplosporellaceae_unclassified_Otu0047 | 0.746655 | 0.012 |
| SRB2_ge_Otu0064 | s__Phoma_multirostrata_Otu0029 | 0.605961 | 0.024 |
| SRB2_ge_Otu0064 | g__Sarocladium_unclassified_Otu0028 | 0.575175 | 0.024 |

1. **Fungi-Fungi**

| Fungi | Fungi | Correlation | p-value |
| --- | --- | --- | --- |
| c__Dothideomycetes_unclassified_Otu0067 | f__Herpotrichiellaceae_unclassified_Otu0098 | 0.576005 | 0.036 |
| c__Dothideomycetes_unclassified_Otu0067 | p__Ascomycota_unclassified_Otu0075 | 0.571979 | 0.024 |
| c__Dothideomycetes_unclassified_Otu0067 | p__Ascomycota_unclassified_Otu0076 | 0.60697 | 0.024 |
| f__Aplosporellaceae_unclassified_Otu0047 | g__Cladosporium_unclassified_Otu0050 | 0.617262 | 0 |
| f__Aplosporellaceae_unclassified_Otu0047 | g__Scopuloides_unclassified_Otu0080 | 0.567295 | 0 |
| f__Aureobasidiaceae_unclassified_Otu0035 | g__Cladosporium_unclassified_Otu0050 | 0.522677 | 0.024 |
| f__Aureobasidiaceae_unclassified_Otu0035 | g__Phacidium_unclassified_Otu0042 | 0.662853 | 0.012 |
| f__Aureobasidiaceae_unclassified_Otu0035 | g__Scopuloides_unclassified_Otu0080 | 0.582008 | 0.024 |
| f__Didymellaceae_unclassified_Otu0011 | f__Didymellaceae_unclassified_Otu0066 | 0.529049 | 0.048 |
| f__Didymellaceae_unclassified_Otu0011 | g__Ramularia_unclassified_Otu0026 | 0.821951 | 0 |
| f__Didymellaceae_unclassified_Otu0011 | g__Sarocladium_unclassified_Otu0028 | -0.505675 | 0.024 |
| f__Nectriaceae_unclassified_Otu0033 | f__Didymellaceae_unclassified_Otu0063 | 0.778865 | 0.012 |
| g__Cladosporium_unclassified_Otu0050 | g__Scopuloides_unclassified_Otu0080 | 0.81354 | 0.012 |
| g__Knufia_unclassified_Otu0055 | c__Dothideomycetes_unclassified_Otu0067 | 0.694715 | 0 |
| g__Knufia_unclassified_Otu0055 | f__Herpotrichiellaceae_unclassified_Otu0098 | 0.862018 | 0.012 |
| g__Knufia_unclassified_Otu0055 | p__Ascomycota_unclassified_Otu0075 | 0.85133 | 0 |
| g__Knufia_unclassified_Otu0055 | p__Ascomycota_unclassified_Otu0076 | 0.905756 | 0 |
| g__Knufia_unclassified_Otu0055 | s__Vishniacozyma_victoriae_Otu0064 | 0.837359 | 0.036 |
| g__Mortierella_unclassified_Otu0002 | f__Didymellaceae_unclassified_Otu0011 | 0.693956 | 0.012 |
| g__Mortierella_unclassified_Otu0002 | g__Ramularia_unclassified_Otu0026 | 0.689547 | 0.024 |
| g__Mortierella_unclassified_Otu0004 | f__Didymellaceae_unclassified_Otu0011 | 0.584143 | 0.048 |
| g__Mortierella_unclassified_Otu0004 | g__Ramularia_unclassified_Otu0026 | 0.609052 | 0.048 |
| g__Mortierella_unclassified_Otu0004 | g__Xylaria_unclassified_Otu0092 | 0.78134 | 0.012 |
| g__Phacidium_unclassified_Otu0042 | f__Aplosporellaceae_unclassified_Otu0047 | 0.626084 | 0.036 |
| g__Phacidium_unclassified_Otu0042 | g__Cladosporium_unclassified_Otu0050 | 0.771077 | 0.012 |
| g__Phacidium_unclassified_Otu0042 | g__Scopuloides_unclassified_Otu0080 | 0.80433 | 0 |
| g__Ramularia_unclassified_Otu0026 | g__Sarocladium_unclassified_Otu0028 | -0.503972 | 0 |
| g__Ramularia_unclassified_Otu0026 | g__Xylaria_unclassified_Otu0092 | 0.593552 | 0.012 |
| g__Sarocladium_unclassified_Otu0028 | s__Phoma_multirostrata_Otu0029 | 0.507955 | 0.024 |
| g__Wallemia_unclassified_Otu0010 | s__Naganishia_friedmannii_Otu0030 | 0.502416 | 0.049 |
| g__Wallemia_unclassified_Otu0010 | s__Sarocladium_bactrocephalum_Otu0056 | 0.714215 | 0.012 |
| k__Fungi_unclassified-Otu0007 | s__Candida_parapsilosis_Otu0107 | 0.898991 | 0 |
| k__Fungi_unclassified-Otu0007 | s__Mycosphaerella_tassiana_Otu0068 | 0.872516 | 0 |
| p__Ascomycota_unclassified_Otu0075 | f__Herpotrichiellaceae_unclassified_Otu0098 | 0.852618 | 0 |
| p__Ascomycota_unclassified_Otu0075 | p__Ascomycota_unclassified_Otu0076 | 0.838526 | 0 |
| p__Ascomycota_unclassified_Otu0076 | f__Herpotrichiellaceae_unclassified_Otu0098 | 0.86109 | 0 |
| s__Debaryomyces_hansenii_Otu0019 | f__Aplosporellaceae_unclassified_Otu0047 | 0.517828 | 0.048 |
| s__Debaryomyces_hansenii_Otu0019 | g__Sarocladium_unclassified_Otu0028 | 0.763854 | 0 |
| s__Mycosphaerella_tassiana_Otu0068 | s__Candida_parapsilosis_Otu0107 | 0.835121 | 0 |
| s__Naganishia_friedmannii_Otu0030 | c__Dothideomycetes_unclassified_Otu0067 | 0.580557 | 0.036 |
| s__Naganishia_friedmannii_Otu0030 | s__Sarocladium_bactrocephalum_Otu0056 | 0.651985 | 0.037 |
| s__Phoma_multirostrata_Otu0029 | f__Aplosporellaceae_unclassified_Otu0047 | 0.616033 | 0.024 |
| s__Phoma_multirostrata_Otu0029 | g__Cladosporium_unclassified_Otu0050 | 0.801348 | 0.013 |
| s__Phoma_multirostrata_Otu0029 | g__Phacidium_unclassified_Otu0042 | 0.76015 | 0.036 |
| s__Phoma_multirostrata_Otu0029 | g__Scopuloides_unclassified_Otu0080 | 0.792529 | 0.012 |
| s__Tremellomycetes_sp_Otu0013 | s__Dioszegia_hungarica_Otu0088 | 0.541452 | 0.025 |
| s__Vishniacozyma_heimaeyensis_Otu0018 | g__Perusta_unclassifiedOtu0083 | 0.545462 | 0.025 |
| s__Vishniacozyma_sp_Otu0023 | g__Perusta_unclassifiedOtu0083 | 0.655715 | 0.012 |
| s__Vishniacozyma_victoriae_Otu0015 | f__Herpotrichiellaceae_unclassified_Otu0098 | 0.75295 | 0 |
| s__Vishniacozyma_victoriae_Otu0015 | g__Knufia_unclassified_Otu0055 | 0.767011 | 0.012 |
| s__Vishniacozyma_victoriae_Otu0015 | p__Ascomycota_unclassified_Otu0075 | 0.78602 | 0 |
| s__Vishniacozyma_victoriae_Otu0015 | p__Ascomycota_unclassified_Otu0076 | 0.788472 | 0.012 |
| s__Vishniacozyma_victoriae_Otu0015 | s__Vishniacozyma_victoriae_Otu0064 | 0.800586 | 0.024 |
| s__Vishniacozyma_victoriae_Otu0064 | c__Dothideomycetes_unclassified_Otu0067 | 0.553255 | 0.012 |
| s__Vishniacozyma_victoriae_Otu0064 | f__Herpotrichiellaceae_unclassified_Otu0098 | 0.863133 | 0 |
| s__Vishniacozyma_victoriae_Otu0064 | p__Ascomycota_unclassified_Otu0075 | 0.839772 | 0 |
| s__Vishniacozyma_victoriae_Otu0064 | p__Ascomycota_unclassified_Otu0076 | 0.85899 | 0 |
| s__Vishniacozyma_victoriae_Otu0015 | s__Vishniacozyma_victoriae_Otu0064 | 0.800586 | 0.024 |
| s__Vishniacozyma_victoriae_Otu0064 | c__Dothideomycetes_unclassified_Otu0067 | 0.553255 | 0.012 |
| s__Vishniacozyma_victoriae_Otu0064 | f__Herpotrichiellaceae_unclassified_Otu0098 | 0.863133 | 0 |
| s__Vishniacozyma_victoriae_Otu0064 | p__Ascomycota_unclassified_Otu0075 | 0.839772 | 0 |
| s__Vishniacozyma_victoriae_Otu0064 | p__Ascomycota_unclassified_Otu0076 | 0.85899 | 0 |

1. **Bacteria-Bacteria**

| Bacteria | Bacteria | Correlation | p-value |
| --- | --- | --- | --- |
| Acholeplasmataceae_unclassified_Otu0022 | Erysipelothrix_Otu0060 | 0.587428 | 0.036 |
| Acholeplasmataceae_unclassified_Otu0022 | Lachnospirales_unclassified_Otu0058 | 0.547895 | 0.012 |
| Acholeplasmataceae_unclassified_Otu0022 | Proteiniclasticum_Otu0059 | 0.799101 | 0 |
| Actinobacteriota_unclassified_Otu0034 | Erysipelothrix_Otu0056 | 0.957135 | 0 |
| Comamonadaceae_unclassified_Otu0016 | Erysipelothrix_Otu0033 | -0.608052 | 0 |
| Comamonadaceae_unclassified_Otu0016 | Erysipelothrix_Otu0060 | -0.597514 | 0.012 |
| Comamonadaceae_unclassified_Otu0016 | Lachnospirales_unclassified_Otu0058 | -0.607966 | 0.012 |
| Comamonadaceae_unclassified_Otu0016 | Soehngenia_Otu0057 | -0.827035 | 0 |
| Dethiobacter_Otu0012 | Acholeplasmataceae_unclassified_Otu0022 | -0.604729 | 0.012 |
| Dethiobacter_Otu0012 | Actinobacteriota_unclassified_Otu0034 | 0.930324 | 0 |
| Dethiobacter_Otu0012 | Dethiosulfatibacter_Otu0017 | -0.753253 | 0 |
| Dethiobacter_Otu0012 | Erysipelothrix_Otu0056 | 0.896727 | 0 |
| Dethiobacter_Otu0012 | Proteiniclasticum_Otu0059 | -0.587777 | 0 |
| Dethiobacter_Otu0012 | SRB2_ge_Otu0023 | 0.5916 | 0.012 |
| Dethiosulfatibacter_Otu0017 | Acholeplasmataceae_unclassified_Otu0022 | 0.800133 | 0 |
| Dethiosulfatibacter_Otu0017 | Actinobacteriota_unclassified_Otu0034 | -0.740097 | 0 |
| Dethiosulfatibacter_Otu0017 | Erysipelothrix_Otu0033 | 0.636169 | 0.012 |
| Dethiosulfatibacter_Otu0017 | Erysipelothrix_Otu0056 | -0.670925 | 0.012 |
| Dethiosulfatibacter_Otu0017 | Lachnospirales_unclassified_Otu0058 | 0.60109 | 0 |
| Dethiosulfatibacter_Otu0017 | Proteiniclasticum_Otu0059 | 0.84788 | 0 |
| Dethiosulfatibacter_Otu0017 | Rhodobacteraceae_unclassified_Otu0018 | 0.592167 | 0.036 |
| Dethiosulfatibacter_Otu0017 | Rhodobacteraceae_unclassified_Otu0035 | 0.613569 | 0 |
| Dethiosulfatibacter_Otu0017 | SRB2_ge_Otu0023 | -0.511492 | 0.024 |
| Erysipelothrix_Otu0033 | Erysipelothrix_Otu0060 | 0.77619 | 0 |
| Erysipelothrix_Otu0033 | Lachnospirales_unclassified_Otu0058 | 0.872154 | 0 |
| Erysipelothrix_Otu0033 | Proteiniclasticum_Otu0059 | 0.832437 | 0 |
| Erysipelothrix_Otu0033 | Soehngenia_Otu0057 | 0.878867 | 0 |
| Erysipelothrix_Otu0033 | SRB2_ge_Otu0063 | 0.608822 | 0.036 |
| Erysipelothrix_Otu0060 | SRB2_ge_Otu0063 | 0.543779 | 0.036 |
| Lachnospirales_unclassified_Otu0058 | Erysipelothrix_Otu0060 | 0.853173 | 0 |
| Lachnospirales_unclassified_Otu0058 | Proteiniclasticum_Otu0059 | 0.884449 | 0 |
| Lachnospirales_unclassified_Otu0058 | SRB2_ge_Otu0063 | 0.650224 | 0 |
| Lachnospirales_unclassified_Otu0058 | SRB2_ge_Otu0064 | 0.569667 | 0 |
| Peptostreptococcales-Tissierellales_unclassified_Otu0006 | Dethiosulfatibacter_Otu0017 | 0.581052 | 0.048 |
| Peptostreptococcales-Tissierellales_unclassified_Otu0006 | Rhodobacteraceae_unclassified_Otu0018 | 0.688549 | 0 |
| Proteiniclasticum_Otu0059 | Erysipelothrix_Otu0060 | 0.834904 | 0 |
| Rhodobacteraceae_unclassified_Otu0018 | Acholeplasmataceae_unclassified_Otu0022 | 0.711473 | 0 |
| Rhodobacteraceae_unclassified_Otu0018 | Proteiniclasticum_Otu0059 | 0.559791 | 0.036 |
| Soehngenia_Otu0057 | Erysipelothrix_Otu0060 | 0.733019 | 0 |
| Soehngenia_Otu0057 | Lachnospirales_unclassified_Otu0058 | 0.83171 | 0 |
| Soehngenia_Otu0057 | Proteiniclasticum_Otu0059 | 0.709292 | 0.012 |
| Soehngenia_Otu0057 | SRB2_ge_Otu0063 | 0.684681 | 0 |
| Soehngenia_Otu0057 | SRB2_ge_Otu0064 | 0.628329 | 0 |
| SRB2_ge_Otu0023 | Actinobacteriota_unclassified_Otu0034 | 0.781481 | 0 |
| SRB2_ge_Otu0023 | Erysipelothrix_Otu0056 | 0.776587 | 0 |
| SRB2_ge_Otu0063 | SRB2_ge_Otu0064 | 0.808705 | 0 |
