# Supplementary material for: Rock Surface Fungi in Deep Continental Biosphere—Exploration of Microbial Community Formation with Subsurface In Situ Biofilm Trap
Source: Microorganisms. 2020 Dec 29;9(1):64. doi: 10.3390/microorganisms9010064 (PMC7824546; doi:10.3390/microorganisms9010064)
Supplement: Supplementary file 1 [file microorganisms-09-00064-s001.zip › Supplementary_figure1.pdf]

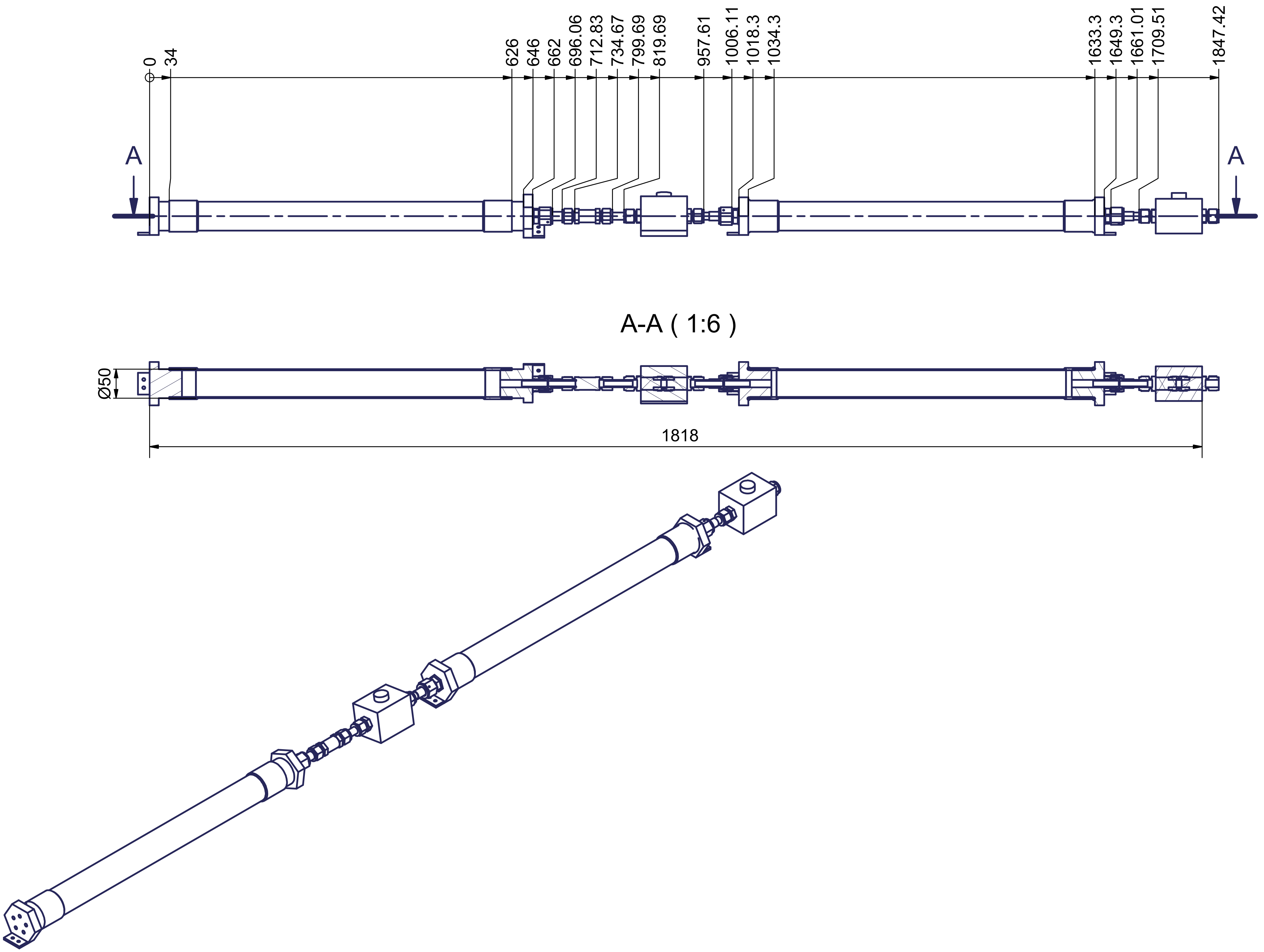
0
34
626
646
662
696.06
712.83
734.67
799.69
819.69
957.61
1006.11
1018.3
1034.3
1633.3
1649.3
1661.01
1709.51
1847.42
A
A
A-A (1:6)
Ø50
1818

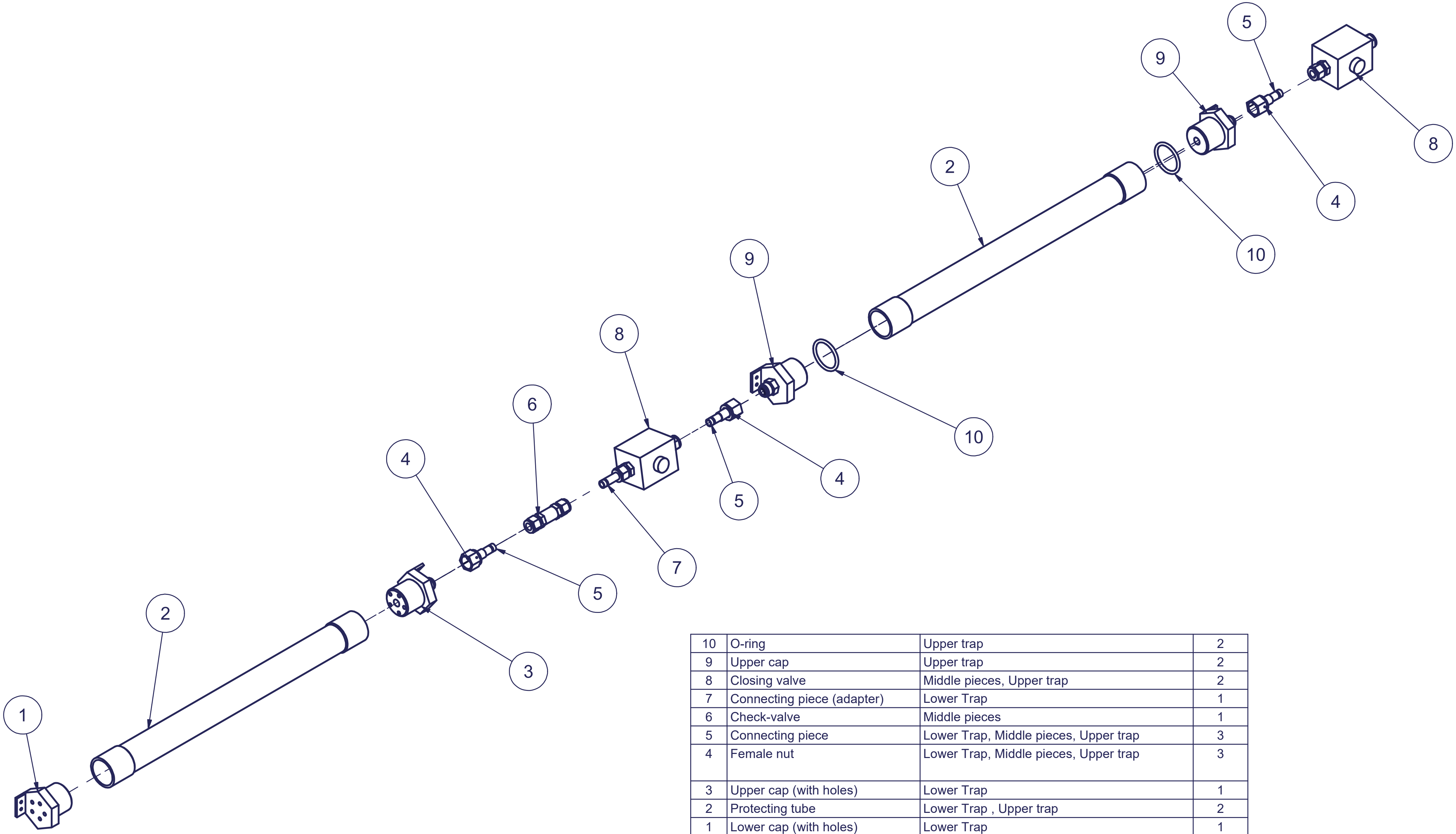

10	O-ring	Upper trap	2
9	Upper cap	Upper trap	2
8	Closing valve	Middle pieces, Upper trap	2
7	Connecting piece (adapter)	Lower Trap	1
6	Check-valve	Middle pieces	1
5	Connecting piece	Lower Trap, Middle pieces, Upper trap	3
4	Female nut	Lower Trap, Middle pieces, Upper trap	3
3	Upper cap (with holes)	Lower Trap	1
2	Protecting tube	Lower Trap , Upper trap	2
1	Lower cap (with holes)	Lower Trap	1
POS	Name	Included in	QTY
